# *LifeSoaks*: a tool for analyzing solvent channels in protein crystals and obstacles for soaking experiments

**DOI:** 10.1107/S205979832300582X

**Published:** 2023-08-10

**Authors:** Jonathan Pletzer-Zelgert, Christiane Ehrt, Inken Fender, Axel Griewel, Florian Flachsenberg, Gerhard Klebe, Matthias Rarey

**Affiliations:** a Center for Bioinformatics, Universität Hamburg, Bundesstrasse 43, 20146 Hamburg, Germany; b Institut für Pharmazeutische Chemie, Universität Marburg, Marbacher Weg 6-10, 35032 Marburg, Germany; Lund University, Sweden

**Keywords:** solvent channels, soaking, *LifeSoaks*, Voronoi diagram, structure-based drug design

## Abstract

Due to the structural complexity of proteins, their corresponding crystal arrangements generally contain a significant amount of solvent-occupied space. These areas allow a certain degree of intracrystalline protein flexibility and mobility of solutes. Therefore, knowledge of the geometry of solvent-filled channels and cavities is essential whenever the dynamics inside a crystal are of interest. Especially in soaking experiments for structure-based drug design, ligands must be able to traverse the crystal solvent channels and reach the corresponding binding pockets. Unsuccessful screenings are sometimes attributed to the geometry of the crystal packing, but the underlying causes are often difficult to understand. This work presents *LifeSoaks*, a novel tool for analyzing and visualizing solvent channels in protein crystals. *LifeSoaks* uses a Voronoi diagram-based periodic channel representation which can be efficiently computed. The size and location of channel bottlenecks, which might hinder molecular diffusion, can be directly derived from this representation. This work presents the calculated bottleneck radii for all crystal structures in the PDB and the analysis of a new, hand-curated data set of structures obtained by soaking experiments. The results indicate that the consideration of bottleneck radii and the visual inspection of channels are beneficial for planning soaking experiments.

## Abstract 2

A novel tool is presented for the calculation of solvent channels and their bottlenecks, with a special emphasis on small-molecule soaking. Bottleneck radii for all crystal structures in the PDB as well as a hand-curated data set of successfully soaked structures are presented.

## 1. Introduction

Determining the 3D structures of large macromolecules is one of the fundamental starting points for our growing biochemical understanding of nature. Even though other methods exist, the most frequently applied experimental method for determining protein or nucleic acid structures is X-ray crystallography (wwPDB Consortium, 2023 ▸), which exploits the signal-amplifying, repetitive arrangement of molecules in a crystal. Besides the determination of new native protein structures, an important application of this technique is the resolution of the poses of small, drug-like molecules bound to proteins in known crystal arrangements. Unfortunately, the crystallization conditions for a specific protein target can be hard to determine in the first place, and it is possible that an additional compound, such as a potential ligand, may prevent the growth of the desired co-crystal (Hassell *et al.*, 2007 ▸). Finding alternative crystallization conditions is often too time-consuming, especially when screening a larger collection of substances to find protein ligands (Wienen-Schmidt *et al.*, 2021 ▸).

Alternatively, the structure can be grown in its native form. The resulting crystal is then transferred into a solution of the molecule of interest. In these soaking experiments, the small molecule to be tested is expected to traverse the previously formed crystal and potentially interact with a corresponding binding site. Soaking is often successfully used to produce protein–ligand complexes and is less time- and material-consuming than co-crystallization studies. Therefore, it is generally used more often, especially in high-throughput (Müller, 2017 ▸) or fragment (Patel *et al.*, 2014 ▸) screening.

However, this technique may fail in some cases because the binding event has to take place in a densely packed crystal environment. Even though on average approximately half of the crystal volume is occupied by solvent, this value can vary from 90% to as low as 25% (Matthews, 1968 ▸; Weichenberger & Rupp, 2014 ▸). Especially for structures with very low solvent content, the natural binding event is potentially hindered or altered (Sanders *et al.*, 2004 ▸; Ehrmann *et al.*, 2017 ▸) or small solvent channels decelerate or prohibit diffusion through the crystal (Geremia *et al.*, 2006 ▸; Nguyen *et al.*, 2021 ▸). Since a known problem during soaking experiments is the potential dismantling of the crystal over time (Ross *et al.*, 2021 ▸), an increased diffusion time can potentially result in false-negative results. As a result, the ligand might not be visible in the X-ray structure even though it would generally bind to the target in solution. Although numerous reports on these problems exist, they only refer to a few exhaustively investigated protein crystal structures (Sanders *et al.*, 2004 ▸; Ehrmann *et al.*, 2017 ▸; Geremia *et al.*, 2006 ▸; Nguyen *et al.*, 2021 ▸). However, many soaking-related false-negative results are probably never detected during the screening of large compound libraries (Hesterkamp & Whittaker, 2008 ▸).

Therefore, it would be desirable to have access to a computational method that detects potential obstacles for soaking experiments in advance. To date, only a few limited ways of estimating the soakability of a crystal have been established. One way is to visually inspect the binding pocket for steric hindrance if its position is known. If the binding site is blocked, co-crystallization or the establishment of conditions that produce a different crystal form can be considered. However, even when the binding pocket seems to be accessible, it is still possible that there is no free path to this site that traverses the crystal (Stum & Gleichmann, 1999 ▸), and it has been shown that size exclusion resulting from narrow or blocked channels is a key factor in the diffusion of small molecules in protein crystals (Cvetkovic *et al.*, 2005 ▸).

A parameter that is often used to estimate the soakability of a system is the Matthews coefficient *V*_M_, which represents the ratio of the crystal volume *V*_a_ per unit of protein mass *M* in the asymmetric unit: *V*_M_ = *V*_a_/*M*. It has a direct inverse relationship to the solvent content of the crystal (Matthews, 1968 ▸). However, the Matthews coefficient does not provide information on the distribution of protein chains in the asymmetric unit. In some cases, large channels traversing the whole crystal might exist even though most of the remaining space is occupied, while in other cases large enclosed areas cannot be reached by any solvate.

To answer the question of whether a small molecule is able to traverse the whole macroscopic crystal and reach the binding pocket, it is necessary to examine the solvent channels in the crystal. However, this task can be challenging since not all crystals harbor linear solvent channels that can easily be located when visualizing several unit cells. Some channels are twisted and curved and therefore hard to capture by eye. Furthermore, these channels may appear to be spacious at some positions but may still include narrow passages representing bottlenecks that hinder or at least decelerate molecular flux inside the crystal.

While the structure of channels inside single proteins can be investigated using various tools such as *MolAxis* (Yaffe *et al.*, 2008 ▸), *Caver* (Chovancová *et al.*, 2012 ▸), *Mole* (Pravda *et al.*, 2018 ▸), *ChExVis* (Masood *et al.*, 2015 ▸) or *BetaCavityWeb* (Kim *et al.*, 2015 ▸), to our knowledge the *MAP_CHANNELS* tool (Juers & Ruffin, 2014 ▸) is the only software that specifically addresses the characterization and visualization of channels in protein crystals. In *MAP_CHANNELS* every point in a grid is annotated by its distance to the closest atom. From this data structure, solvent channels are analyzed to yield various descriptors such as bottleneck radii, tortuosity, width variation and anisotropy. To this end, a connected neighbor connectivity search with respect to a fixed cutoff is performed. Consequently, channels have to be calculated multiple times for different radii.

Unfortunately, grid approaches have the inherent dis­advantage that they are not invariant to axis rotations and that the number of grid points scales cubically with the inverse of the grid spacing *d*. Furthermore, the roughly linear relation between atom count *N* and unit-cell volume *V* results in an asymptotic run-time behavior that lies in Θ(*N* ^2^ · *d* ^−3^), since the distance of all grid points, the count of which lies in Θ(*V* · *d* ^−3^), is compared with every atom of the unit cell. With an increasing number of atoms, the run time can become very high, which may require an increase in the grid spacing for some cases with large unit cells, leading to more coarse-grained models.

In this work, we aim to address the problem of reliable and efficient crystal channel analysis and subsequent soakability predictions with our new tool *LifeSoaks*. *LifeSoaks* was designed to provide an intuitive way to visually inspect solvent channels in crystals and to enable the analysis of channel bottlenecks for large structure sets. Special focus was given to the periodic nature of a crystal to guarantee that a channel in a unit cell always corresponds to a crystal-traversing path on the macroscopic level.

The method calculates so-called local bottleneck radii. For a given position *p*, the local bottleneck radius represents the maximal radius that a sphere may have in order to reach *p*.

For a given structure in PDB or mmCIF format with a valid CRYST1 entry, *LifeSoaks* calculates bottleneck radii for the solvent-occupied space, represented by a set of spheres, and stores it locally. Consequently, any position that is part of such a sphere has a local bottleneck radius. Thereby, channels can be visualized by displaying all space with a radius larger than or equal to any user-defined cutoff. Additionally, it can determine the bottleneck radius for the main solvent channel as well as channels leading to binding pockets. By using a Voronoi diagram-based approach, as has become standard in single protein-channel prediction tools, we can do so efficiently and account for the macroscopic periodicity.

We will present and discuss the bottleneck radii for all publicly available crystal structures in the Protein Data Bank (PDB; Berman *et al.*, 2000 ▸) and a new curated data set of protein–ligand crystals that were obtained by soaking experiments. Note that although *LifeSoaks* can handle nucleic acid structures, we will refer to the handled molecules as proteins in the following for simplicity. *LifeSoaks* is made available as a standalone tool and as part of the Proteins*Plus* web server (https://proteins.plus).

## 2. Methods

The question at hand is whether a small molecule can traverse a crystal and reach a potential binding site. We address this problem by determining the free space that an object, *i.e.* a ligand, may reach in the crystal on a periodic path through solvent channels. To this end, we need to identify and characterize the solvent-occupied space in the crystal.

In the context of this method, this highly complex question will be handled by a geometric approach that approximates the 3D shape of a ligand by a sphere. Based on this assumption, we want *LifeSoaks* to provide information as to how large a sphere may be at maximum in order to reach a position of interest in the crystal and to visualize the accessible space.

In the context of a crystal channel, this task consists of two main problems. Firstly, the detected paths must maximize the minimal distance to protein atoms since the radius of the sphere is determined by the narrowest subpart of the path. Secondly, the path needs to be periodic to correspond to an actual path in the macroscopic crystal.

To this end, the *LifeSoaks* algorithm calculates a Voronoi diagram for one complete unit cell. From this, we construct a reduced Voronoi channel graph that satisfies the periodic boundary condition by including artificial edges connecting the surfaces and thereby enables the detection of periodic paths. The final channel detection is not performed by computing explicit paths but by using a set-based approach that assigns the maximum radius that a sphere may have to reach it on a periodic path to each vertex. Channels are thereby defined by a dynamic cutoff and include all vertices with a radius greater than or equal to this cutoff. Since this method stores bottleneck radii locally, positions of interest, such as a binding site, can easily be analyzed.

### 2.1. The *LifeSoaks* algorithm

#### 2.1.1. Describing crystal solvent channels with Voronoi diagrams

The problem of finding a path for the largest possible sphere is strongly related to the medial axis of the channel, which can be described as the subspace for which more than one closest point to the channel boundary exists (Blum, 1967 ▸). The medial axis includes all space where the center of maximal spheres can be placed, since a sphere touching only one boundary can still be enlarged in another direction.

In *LifeSoaks*, we use the closely related concept of a Voronoi diagram to describe the solvent-occupied space (Voronoi, 1908 ▸). For a collection of points, it can be shown that all corresponding Voronoi objects are also part of the medial axis (Fabbri *et al.*, 2002 ▸). However, unlike the medial axis, a 3D Voronoi diagram can be calculated in expected 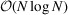 in typical cases (Boissonnat & Attali, 2002 ▸; Amenta *et al.*, 2007 ▸), even though the worst-case complexity is 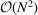 (Dewdney & Vranch, 1977 ▸). To compute the Voronoi diagram, we used an in-house implementation of a 3D incremental insertion algorithm to construct a Delaunay tetrahedralization (Green & Sibson, 1978 ▸; Guibas & Stolfi, 1985 ▸; Joe, 1991 ▸). A Delaunay tetrahedralization is dual to a Voronoi diagram. We can subsequently generate the Voronoi diagram in Θ(*N*) time from the tetrahedralization.

A Voronoi diagram in *d* dimensions computed on a set of input points *P* with 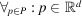 assigns the subspace *S*_*p*_ to every point *p* for which the following relation holds:

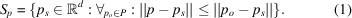

**Figure 1:**
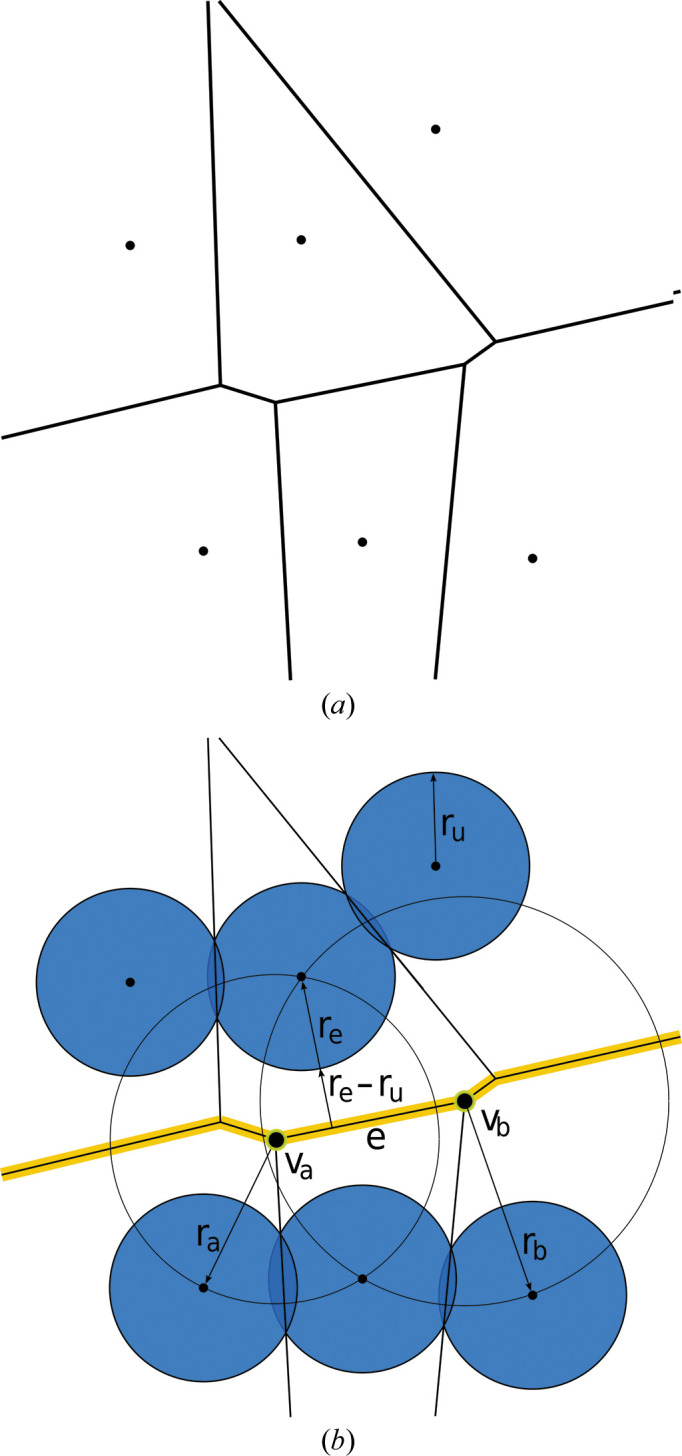
(*a*) An example of a two-dimensional Voronoi diagram. The lines are parts of the perpendicular bisectors of the neighboring points and are good approximations for paths that maximize the minimal distance to points. (*b*) The same Voronoi diagram in a molecular interpretation. The points represent the atoms depicted in blue. The lines are paths with maximal distance to atom centers. The yellow line marks a potential channel with a certain minimum distance to the atoms. The edge radius is illustrated for edge *e* = (*v*_*a*_, *v*_*b*_). *r*_*e*_ is the minimal distance of *e* to its closest atom center. For a molecular representation, *r*_*e*_ is reduced by *r*_u_, resulting in the minimal distance to an atom surface.

Thus, *S*_*p*_ is the space for which *p* is the closest of the points in *P*. The resulting structure consists of convex polyhedra around every point *p* (see Fig. 1 ▸ *a* for a 2D example). Their facets represent areas at which another point is equally distant, edges represent areas at which two additional points are equally distant and vertices are equally distant to four elements of *P* in total. The vertices and edges of the Voronoi diagram can be interpreted as a graph with an annotated radius of free space at every graph element. For vertices, this radius represents the distance to its four defining/closest elements in *P*. For edges, it is the minimal distance to its three defining elements and the radius is calculated from their planar circumcircle. The circumcircle also represents the intersection of the spheres of free space surrounding the vertices incident to the edge. The space occupied by all of these spheres represents our model of the solvent-occupied space.

Constructing a Voronoi diagram from a molecular structure poses additional challenges in comparison to the general case. One of them is that we need to treat atoms as spheres for an adequate representation. However, since Voronoi diagrams in their standard form are generally defined on points, the available algorithms can only treat atoms as spheres of equal radius (see Fig. 1 ▸ *b*). This limitation is sometimes addressed by approximating large atoms by several spheres (Yaffe *et al.*, 2008 ▸; Chovancová *et al.*, 2012 ▸) or even by sophisticated Euclidean Voronoi diagrams that treat arbitrary spheres explicitly, leading to curved Voronoi elements (Kim *et al.*, 2005 ▸, 2015 ▸).

Since calculating Voronoi diagrams for a unit cell is computationally expensive, *LifeSoaks* uses a standard implementation with a unified sphere radius *r*_u_, which will be subtracted from all vertex and edge radii. By default, *r*_u_ is set to the van der Waals radius of a carbon atom (1.7 Å; Bondi, 1964 ▸). While this only slightly overestimates the 1.5 Å radius of oxygen and nitrogen atoms, hydrogen atoms, with a van der Waals radius of 1.1 Å (Rowland & Taylor, 1996 ▸), are significantly smaller. Therefore, the corresponding spheres are moved *r*_u_ − 1.1 Å towards the connected heavy atom to decrease the overestimated space. In the common case that hydrogen atoms are not assigned in the PDB file, they will be added by our chemistry model with respective binding geometries beforehand (Urbaczek *et al.*, 2013 ▸).

Another challenge is the common local coplanarity of substructures originating from symmetries or planar ring systems. Four atoms placed exactly on the same plane cannot form a tetrahedron, which may cause the algorithm to either fail or run for unfavorably long. This problem is addressed by perturbing the position of input points randomly within a 10^−6^ Å radius around their origin.

In an effort to reduce the computation time, the solvent-accessible surface (SAS) area is determined prior to calculation by the in-house implementation of a spherical-probe SAS algorithm (Richards, 1977 ▸; Reulecke *et al.*, 2008 ▸). Atoms that are not solvent-exposed are discarded since they cannot be part of a solvent-channel boundary.

By default, small molecules and solvents such as water are not considered in the calculation and are treated as empty space. However, the user can include such molecules explicitly, for example to consider binders with high residence times. Like the protein itself, they are regarded as completely rigid since the model does not consider flexibility.

To represent entire crystal solvent channels by Voronoi elements, we need to construct a Voronoi diagram of one complete unit cell. Since a crystal can be constructed from one unit cell by applying translation operations, the same holds for its solvent channels. However, every element of the Voronoi diagram is defined by its closest atoms. Therefore, it is necessary to account for any atoms of neighboring unit cells that reach into the central unit cell or are close enough to the border to influence the outer Voronoi elements. This was achieved by adding additional symmetry copies at the unit-cell borders. Roughly half an additional unit cell in each direction and dimension is necessary to correctly compute the central unit cell, resulting in an input of approximately eight unit cells (see the supporting information for a detailed derivation of the space depending on the unit-cell geometry).

Since we are interested in a Voronoi diagram of one complete unit cell, the Voronoi diagram calculated from this input is subsequently cut at the borders of the central unit cell and artificial surface vertices are introduced at the cutting positions of edges. Furthermore, Voronoi elements are discarded if their radius is smaller than a user-defined cutoff since they are considered to be irrelevant for detecting solvent channels while inflating the resulting structure. Note that these artificial alterations result in a structure that is no longer an actual Voronoi diagram; therefore, the resulting graph will be referred to as Voronoi channel graph in the following.

#### 2.1.2. Accounting for crystal periodicity in a Voronoi channel graph

In general, we are interested in calculating paths that traverse a macroscopic crystal consisting of numerous unit cells. Therefore, the paths that we calculate not only need to reach specific positions in the unit cell but also need to do so in a periodic manner. Starting from a crystal or unit-cell surface, the path, in theory, needs to reach infinitely many subsequent unit cells.

**Figure 2:**
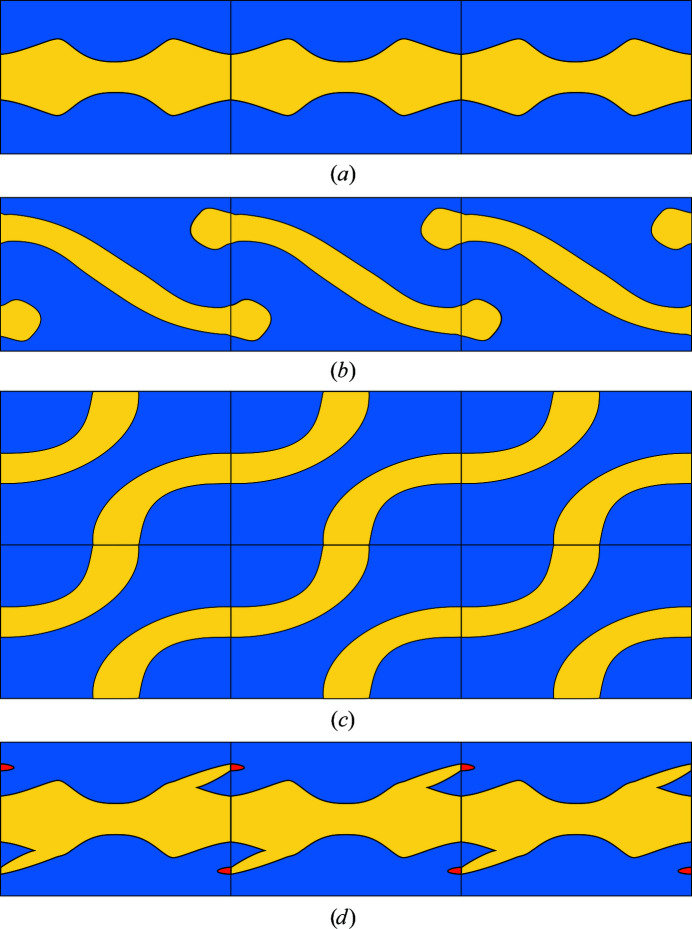
Four exemplary channel geometries that pose different requirements for the model. The channels are shown in yellow, while protein-occupied space is depicted in blue. (*a*) A trivial channel that linearly passes one unit cell before reaching its periodic starting point. (*b*) A channel that traverses the whole unit cell. Since its surfaces do not map onto each other, it does not traverse the macroscopic crystal. Therefore, it never reaches its starting point and cannot be detected by the model. (*c*) A channel that traverses the whole unit cell but crosses a second unit-cell boundary before reaching its starting position. These cases can be detected by considering the periodic boundary condition. (*d*) A special case that leads to the region marked in red not being correctly handled by the model.

Such paths are trivial to detect in some cases, for example when the channel traverses a single unit cell and ends at its periodic starting point (Fig. 2 ▸ *a*). However, as shown in Fig. 2 ▸(*b*), this condition is not always met. Even though the depicted channel traverses a complete unit cell, it is interrupted right after crossing the boundary to the next one and cannot reach its periodic starting point. These cases can be detected by projecting the intersections of opposing unit-cell surfaces onto each other and checking for overlap.

In other cases, channels may traverse several unit cells before reaching their periodic starting point (Fig. 2 ▸ *c*). Here, the Voronoi channel graph must accurately represent the periodic condition in the channel construction.

Therefore, we introduced artificial Voronoi edges that connect surface vertices directly in front of each other on opposite sides of the unit cell. On the macroscopic level, these edges represent connections to the neighboring unit cell. Subsequently, channels can be computed in full even if they cross the boundaries at some positions. A channel that exits the unit cell re-enters it on the opposite side when treated as passable.

A further special case in the model is shown in Fig. 2 ▸(*d*). It can occur when enclosed side areas of a channel are cut by the unit-cell boundary and these areas have no other connection to the main channel. In the algorithm, the unit-cell surface currently checked for channels has to be treated as non-passable, since otherwise the erroneous detection of local circles would be possible. Therefore, these special regions are isolated even though they are connected to the channel. In all cases visually inspected in the context of this study, this scenario had no significant impact.

#### 2.1.3. Calculating bottleneck radii for channels

In general, the Voronoi channel graph may include a large number of paths from one crystal surface to the opposite surface. To predict the ability of molecules to traverse the crystal, we define the quality of each path by its narrowest subpart, *i.e.* its bottleneck radius. This decision leads to a particular difference from several previously mentioned Voronoi-based tools for detecting single protein channels, which often determine the optimal path using a shortest-path algorithm. Their distance measure often includes an inverse contribution of the local radius. However, the resulting optimal path also depends on its length, which we wanted to avoid.

Instead of computing the shortest paths, we define channels with respect to an arbitrary radius *r*′ as a subset of Voronoi vertices 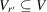 that can be reached by a sphere with radius *r*′ on an infinite periodic path through the crystal.

Let *I*_*P*_ be the set of all infinite crystal paths that periodically traverse subsequent unit cells, thereby describing a path through the macroscopic crystal. For a given vertex *v* the following relation holds:

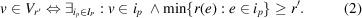

Thus *v* is reachable by a sphere of radius *r*′ if and only if there exists an infinite path in the Voronoi channel graph that contains *v* while the radius *r*(*e*) of its thinnest edge is at least *r*′.

The *LifeSoaks* algorithm aims to annotate each vertex with its local bottleneck radius *r*_b_(*v*), for which the following relation holds:

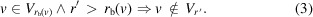

Thus *r*_b_(*v*) is the largest bottleneck radius of all paths that include *v*. This radius may be much lower than the local vertex radius *r*(*v*), for example if *v* is part of a large enclosed cavity that can only be reached by a small channel. However, *r*_b_(*v*) can never exceed *r*(*v*).

**Figure 3:**
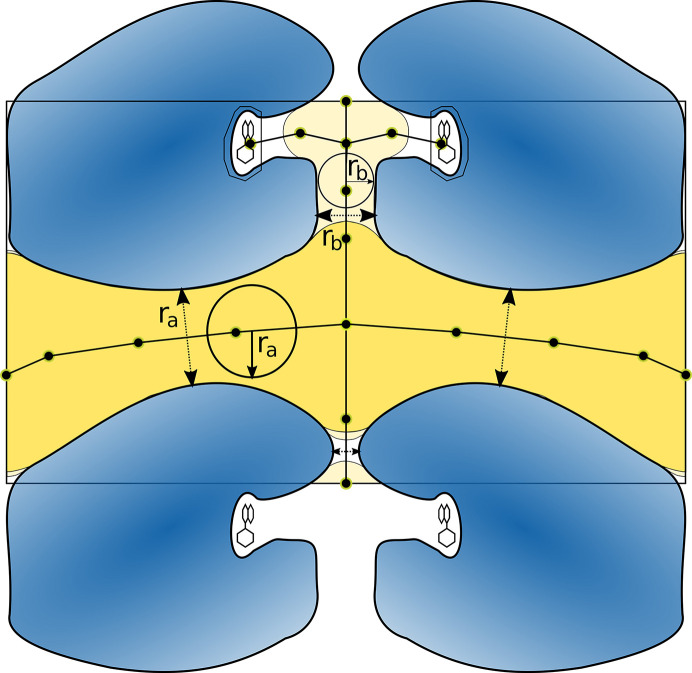
A two-dimensional schematic of a crystal unit cell with a simplified Voronoi channel graph. Blue objects are proteins with a ligand in a binding site. The double arrows mark bottleneck positions. Bottleneck *a* with radius *r*_*a*_ defines the space visualized in dark yellow that a sphere of radius *r*_*a*_ or smaller can reach. Bottleneck *b* has radius *r*_*b*_. It defines the space marked in light yellow. Since *r*_*b*_ < *r*_*a*_, a sphere of radius *r*_*b*_ can also be placed in all of the dark yellow space. The white space cannot be reached by either of the two spheres but may potentially be reached by smaller spheres. A typical unit-cell Voronoi diagram consists of thousands of edges and vertices. The exact number can optionally be reported in the output.

*r*_b_(*v*) can be stored locally at each vertex. A channel set 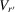 is derived by including every vertex with *r*_b_(*v*) ≥ *r*′. The principle is visualized in Fig. 3 ▸.

**Figure 4:**
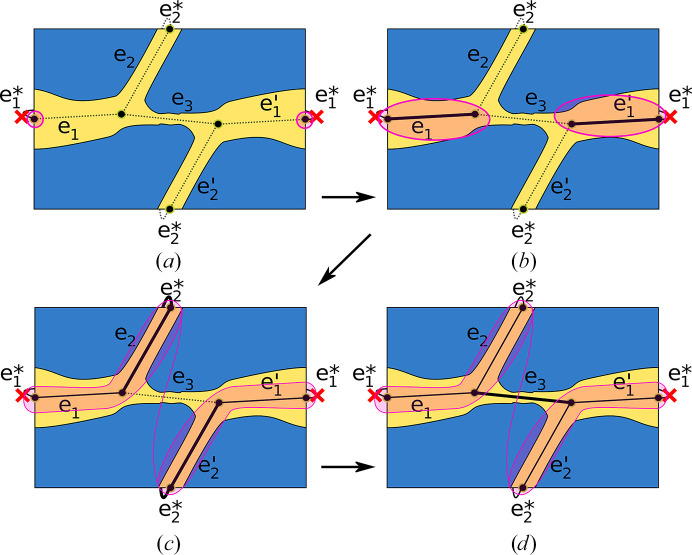
A two-dimensional schematic of the *LifeSoaks* algorithm. The unit cell includes two internal vertices, four surface vertices, five internal edges and two artificial surface-connecting edges marked *. We note that 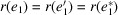 and 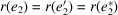 due to internal point symmetry. Further, *r*(*e*_1_) > *r*(*e*_2_) > *r*(*e*_3_). (*a*) The state before any edge is processed. In this iteration the bottleneck radii are determined from left to right, so vertices on these sides are marked as surface vertices (magenta coloring) and 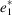 is excluded. (*b*) After two iterations *e*_1_ and 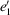 have been processed, since they are the largest edges. Because they connect one new vertex to a surface vertex, their incident vertex is now also accessible by one surface but not by both. (*c*) After three additional iterations *e*_2_, 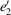 and 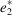 have been processed. Since 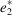 connects the surface vertices at the top and bottom, all vertices are now part of the same set. Since this set now contains vertices of the opposing surfaces, it represents a unit-cell-traversing path and all vertices will be annotated with the bottleneck radius *r*(*e*_2_). (*d*) Finally, *e*_3_ is processed. However, because the incident vertices are already part of the same set, no set or radius is changed.

This concept is realized by implementing an algorithm that is based on a union–find data structure that saves disjoint sets of Voronoi vertices in a new directed tree graph. Finding the set of a vertex as well as combining two sets into one can be efficiently performed in 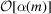 , where *m* is the number of set elements and α denotes the inverse of the Ackermann function, which can be considered to be constant for practical inputs (Ackermann, 1928 ▸; Galler & Fisher, 1964 ▸; Tarjan & van Leeuwen, 1984 ▸). The pseudocode is a simplification of the main routine, neglecting several implementation details such as optimizations. A schematic visualization of the main steps of the algorithm can be found in Fig. 4 ▸.

The algorithm is performed three times, once for every unit-cell dimension/axis (line 3 in the pseudocode shown below). In each iteration, one pair of opposing unit-cell surfaces is checked for paths through the entire unit cell. Therefore, artificial surface-connecting edges are not included in the calculation for this surface pair (line 7). The other two surface plane pairs are treated as passable to detect channels, as depicted in Fig. 2 ▸(*c*).

To begin with, every Voronoi vertex is a single set. Surface Voronoi vertices of the two considered planes are labeled accordingly. Afterwards, all Voronoi edges are processed in descending order with respect to their radii (lines 2 and 5). Whenever an edge connects two previously disjoint union–find sets, these sets are combined (line 9) and whether this set contains a path through the unit cell is checked. This holds if one of two conditions is met. If one set already includes a complete path, this path is now accessible to the union of both sets. However, if neither set includes a previously discovered path, it is checked whether they reach opposing sides of the considered surface pair and whether these surface vertices can be projected onto each other (line 16). In both cases the union represents an infinite path through the crystal and the radius of the current edge is annotated to every included Voronoi vertex unless it had previously been assigned a larger radius (lines 17–20). The radius-descendent edge processing ensures that a connected path is always found exactly when its narrowest edge is included, automatically delivering its bottleneck radius for that dimension.

Ultimately, every vertex is annotated with a radius for each unit-cell dimension, the largest of which represents its bottleneck radius. For a specific vertex *v*, this radius *r*_b_(*v*) corresponds to the radius of the largest spherical object that can reach this vertex on an infinite periodic path through the crystal. The largest local bottleneck radius among all vertices corresponds to the bottleneck radius of the main channel of the crystal. Note that separate bottleneck information for all three dimensions can also be of interest since the blocking of diffusion directions can be associated with significantly increased soaking times (Geremia *et al.*, 2006 ▸). 
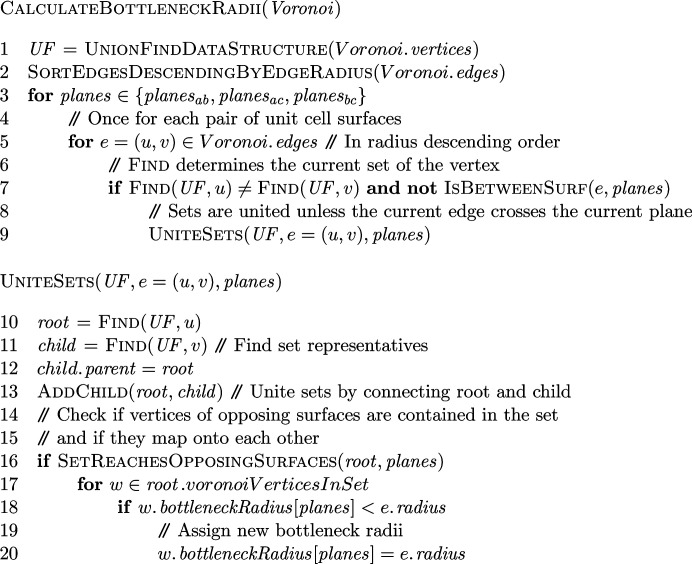


#### 2.1.4. Assigning bottleneck radii to binding pockets

To describe the solvent-channel path with respect to a binding pocket, we introduced a routine that assigns a bottleneck radius to user-defined binding sites.

In our code base, binding sites are either derived from a unique ligand identifier by defining every residue within a 6.5 Å radius as part of the pocket or directly provided by additional PDB files containing the binding-site residues. The convex hull for all atoms of pocket residues is calculated and defined as the pocket space.

If the pocket space contains Voronoi elements, a local bottleneck radius could be extracted from this information alone. However, the complex binding event is not well described by our simplified representation. Instead, we want to predict whether the ligand can reach the space in front of a binding pocket. Therefore, the Voronoi channel graph is searched for the largest bottleneck radius *r*_b,poc_ within a user-defined distance of the convex hull of the pocket. After determining *r*_b,poc_, a breadth-first search is performed on the Voronoi channel graph to locate the corresponding bottleneck position, *i.e.* the first edge *e* = (*u*, *v*) for which *r*(*e*) = *r*_b,poc_ = *r*_b_(*u*). Note that this method may fail if the binding site is not pocket-like since it might contain no Voronoi elements. Observed failures can usually be attributed to flat convex hulls of binding sites on the protein surface.

#### 2.1.5. Data representation and visualization with *LifeSoaks*

*LifeSoaks* assigns a bottleneck radius to every Voronoi vertex and to the crystal structure as a whole. To allow dynamic visualization of channels considering all local bottleneck radii, *LifeSoaks* writes grid files in the CCP4 format (Winn *et al.*, 2011 ▸) by converting the annotated Voronoi channel graph into a grid. Each grid point is annotated by the local bottleneck radius of the largest vertex sphere containing it. Various molecular-visualization programs such as *UCSF Chimera* (Pettersen *et al.*, 2004 ▸), *PyMOL* (Schrödinger) and *Coot* (Emsley *et al.*, 2010 ▸) support this format and display the grid data with respect to user-defined cutoff values. Note that for accurate channel representation the true values have to be visualized and not the standard deviation σ of the grid, which is commonly used for electron-density maps. For a specific radius, the displayed grid volume represents the space in which a sphere of that radius could roll around freely on an infinite path through the crystal. The radius represents *r*′ and the visualized grid resembles the corresponding 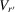 as described in formula (2).

With respect to a position of interest, such as a binding pocket, the user may gradually decrease *r*′ until the channel reaches that position.

In addition to the standalone command-line tool, the functionality is freely available on the Proteins*Plus* web server at https://proteins.plus (Fährrolfes *et al.*, 2017 ▸; Schöning-Stierand *et al.*, 2022 ▸).

Starting with *LifeSoaks* version 1.1, the command-line tool will also include an option for two additional types of grid visualization. One contains the true local radius at each position, so a sphere with the current cutoff radius may be placed anywhere inside the grid but can potentially not move around freely. The other contains the local distance to the closest atoms. The displayed grid represents the space in which the center of the potentially blocked sphere can be placed.

#### 2.1.6. Matthews coefficient and solvent-content calculation

To compare the bottleneck radii with the crystal solvent content and the Matthews coefficient, we did not use the values deposited in the PDB since we discovered several inconsistencies in the deposited values. We suspect that slight differences in the various software tools used for crystallo­graphic structure determination are the cause of this. We recalculated these values from the protein mass *M*_a_ of our internal chemistry model (Urbaczek *et al.*, 2011 ▸, 2012 ▸) to obtain consistent results. The solvent content is calculated using an average protein density δ of 0.81 Da Å^−3^ as described by Matthews (1968 ▸). It is then expected to be the volume not occupied by protein *p*_solv_ = 1 − *p*_prot_ = 1 − [*M*_prot_/(δ × *V*_a_)]. *V*_a_ represents the volume per asymmetric unit. Note that, for the sake of simplicity, the calculation is not altered for nucleic acids, which are generally assumed to have a lower density (Kantardjieff & Rupp, 2003 ▸).

#### 2.1.7. Implementation and computational requirements

*LifeSoaks* was written in C++ as part of the NAOMI cheminformatics library (Urbaczek *et al.*, 2011 ▸, 2012 ▸). The computation of the Delaunay tetrahedralization, which is not parallelized, takes the major part of the computation time and storage. The conversion into a Voronoi channel graph and the union–find operations of the channel-computation routine can be neglected in comparison. The tool typically requires a few minutes for one crystal structure on a standard desktop machine. Longer computation times occasionally occur for large structures with complex crystal symmetries or due to unfavorable input distributions. Cases with Ω(*N*log*N*) tetrahedra also lead to high memory requirements. *LifeSoaks* has an included heuristic memory limitation which terminates the calculation at a user-defined memory usage to prevent memory overflows. This is set to 6 GB by default but needs to be increased for large structures.

### 2.2. Benchmarking *LifeSoaks*

To test the benefit of *LifeSoaks*, we need to compare its results with the outcomes of soaking experiments. Ideally, we would test the ability of *LifeSoaks* to predict unsuccessful experiments on a large scale correctly. Unfortunately, documented problems during soaking in the literature are anecdotal and insufficient for a robust benchmark data set. Instead, our data set consists of true-positive cases, *i.e.* protein–ligand structures that were successfully solved by soaking, to assess the reliability of prediction.

#### 2.2.1. Data-set generation

We designed a data set of protein–ligand complex structures that were obtained by soaking a ligand-free crystal in a solution containing the ligand. To this end, we screened the REMARK 280 lines of all X-ray crystallographic structures in the PDB. These remark lines contain experimental details of the crystallization conditions. Structures containing the string ‘soak’ (case-insensitive) in this remark were analyzed. Based on the description of the crystallization procedure in the PDB file, we built a data set including the PDB codes and the HET codes of the soaked ligands. We extracted the experimental details from the associated publication whenever the description was ambiguous. Finally, we split the data set into two subsets: (i) soaked small molecules, ions and atoms (‘Ligand-Soaked Complexes’) and (ii) soaked oligomeric or polymeric compounds, *i.e.* oligosaccharides, peptides, proteins or nucleic acids (‘Oligomer-Soaked Complexes’). The second subset was not used in the subsequent analyses as the reliability of conformer predictions for oligomeric or even polymeric molecules with many rotatable bonds has limitations (Friedrich *et al.*, 2017 ▸), leading to poor interpretability of the results. The first subset used in our analyses comprised 1713 structures and 4718 ligands that have been reported to have been soaked into preformed crystals. Whenever these ligands were found in the structure, we considered all corresponding ligands and their binding sites. For ligand detection, we used an in-house tool developed with our NAOMI library (Urbaczek *et al.*, 2011 ▸, 2012 ▸). All entities identified as ligand molecules and residues listed in the HET section of the PDB files are checked for the HET code of the soaked molecule. The ligand instances with this HET code were considered in soaking predictions. The full data set, including the PDB codes and ligand identifiers, is readily available via the GitHub repository for the project (see the Supporting information).

#### 2.2.2. Ligand radii assignment for ligand-soaked complexes

Since our geometrical model describes the objects traversing a channel by a single radius, we need a procedure to determine a meaningful radius for a given molecule conformation. We first predict the most probable protonation state of each ligand from its SMILES representation with *UNICON* (Sommer *et al.*, 2016 ▸; Sommer, 2016 ▸). The protonation and tautomer-generation mode was set to single, *i.e.* only the top-scoring states are used. Subsequently, the conformer-generation method *Conformator* (Friedrich *et al.*, 2019 ▸; Friedrich, 2019 ▸) was applied to generate at most 1000 conformations per ligand with default parameters. Both tools are freely available for noncommercial and academic use and can be found using the links in the corresponding references. For seven ligands (PDB ligand IDs AF3, BEF, DQY, PA0, SEY, VM4 and WO4), preparation of the ligand with *UNICON* failed and only their ideal conformation as downloaded from the PDB was used. However, for one ligand (ligand ID 6VQ) the workflow was successfully performed with the respective input in SDF format with ideal coordinates from the PDB but not with SMILES.

Based on the number of generated conformations, we split the set into molecules with a single conformer and molecules with multiple conformers. The minimum projection radii of all conformers were subsequently calculated using the *ChemAxon* tool *cxcalc* (https://www.chemaxon.com). Based on the van der Waals radii of the molecule atoms, the method searches for the enclosing circular planes onto which all atoms can be projected. It reports the radius of that with the smallest radius (the minimum projection radius). For single atoms (for example xenon) and ions that could not be annotated with their respective projection radii, we referred to two publications reporting experimentally determined and calculated radii (Shannon, 1976 ▸; Rahm *et al.*, 2016 ▸). The assigned projection radii can be found in the GitHub repository for the project.

#### 2.2.3. Software parameters

*LifeSoaks* runs on the hand-curated benchmark data set were performed with the default settings apart from the following changes: the minimum channel radius was adjusted to 0.5 Å if the minimum projection radius of a ligand was below the default of 1.7 Å and the memory limit was set to 120 GB. None of the calculations for the data set exceeded this limit. Protein structures in PDB format were used to reproduce the behavior of the tool on the Proteins*Plus* web server. PDB structures with multiple models lead to software failures and should be preprocessed by the user to include only one model. *MAP_CHANNELS* runs were performed with the default settings apart from the following changes: we enforced the use of the specified grid size of 2.0 Å regardless of the maximum time. In addition, we set the channel cutoff, *i.e.* the minimum grid–atom surface distance to be considered a channel, to 0.5 Å.

#### 2.2.4. *EDIA* calculations

Since we work with a rigid protein model but expect that highly flexible residues do not represent a strict obstacle for soaked molecules, we performed a second calculation in which we excluded parts of the structure that are poorly supported by electron density. To this end, the electron-density score for individual atoms that quantifies the electron-density fit of an atom was calculated with *EDIA* (Meyder *et al.*, 2017 ▸). If available, the corresponding 2*F*_o_ − *F*_c_ electron-density maps were downloaded from the PDBe (Armstrong *et al.*, 2020 ▸). For structures with available electron-density maps, *EDIA* was applied with default parameters. Residues with at least four atoms with an *EDIA* score below 0.8 were excluded from the solvent-channel calculations with *LifeSoaks* in this additional run.

### 2.3. Plotting and data preparation

The results were preprocessed with the Python packages *pandas* (McKinney, 2010 ▸), *NumPy* (Harris *et al.*, 2020 ▸) and *SciPy* (Virtanen *et al.*, 2020 ▸). Plotting was performed using *Matplotlib* (Hunter, 2007 ▸) either directly or using the *seaborn* library (Waskom, 2021 ▸).

## 3. Results and discussion

### 3.1. Calculating channel bottlenecks for the whole PDB

Since *LifeSoaks* can efficiently calculate bottleneck radii for protein crystals without manual intervention, we performed this analysis for each structure deposited in the PDB. At the time of analysis (18 August 2022) 193 452 structures had been deposited, of which we could process 193 448. 25 571 structures originate from crystal-independent methods such as cryo-EM or NMR. Therefore, they have no meaningful CRYST1 entry and were discarded, leaving 167 877 structures. 53 structures were discarded because they contained no atoms. This is caused by very small structures being classified as ligands by our chemistry model. The resulting 167 824 structures were used as input for *LifeSoaks*, with default parameters of 1.7 Å for the unified atom radius and 1.7 Å for the minimum radius. Note that these structures include many duplicates, especially for intensively investigated proteins with many PDB-deposited structures. However, since the unit-cell geometry can vary with differing experimental conditions, we decided not to exclude them. The memory limit was set to 12 GB. This was sufficient for 155 483 structures for which valid results could be created. The remaining 12 341 structures are predominantly characterized by a large number of atoms per unit cell, which is caused by a large protein, numerous symmetry copies of the asymmetric unit or a combination of both. By repeating the experiment with a memory limit of 60 GB, 11 532 more cases were computed successfully, while 393 additional structures were only successful when increasing the limit to 120 GB. 416 cases failed in every computation. Most of these are extremely large structures such as ribosomes or virus capsids that probably require even more memory. Other failure cases may be caused by unresolved coplanarity or small structures packed so densely that they do not include Voronoi elements larger than the minimum radius.

**Figure 5:**
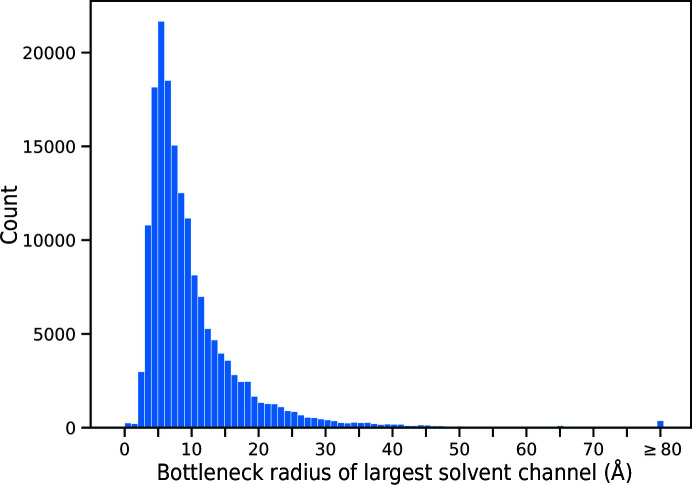
A histogram of the bottleneck radius frequencies in crystal structures from the PDB. Each bin represents exactly 1 Å, except for the last bin, which includes all structures with a bottleneck radius greater than 80 Å.

In total, the successful computations include 166 408 structures. Fig. 5 ▸ shows the distribution of the bottleneck radii for their largest channels. For all but 253 structures, channels above the 1.7 Å threshold could be found. The mean bottleneck radius is 10.3 Å, while the median is 7.7 Å. Although the largest channels will not always describe paths that can reach binding sites, they can, to a certain degree, describe how large molecules that enter the crystal may be. In extreme cases, even whole proteins may be soaked into the crystal (Hashimoto *et al.*, 2019 ▸). Our analysis determined 371 structures with bottleneck radii of at least 80 Å for which this seems possible. On the other hand, 14 676 structures (8.8%) exhibit bottleneck radii of less than 4 Å. This radius is of the order of magnitude of a benzene ring and potentially hinders the flux of drug-like molecules. However, it has to be kept in mind that the simplified radius description may not be adequate if the intersection of the bottleneck is closer to an ellipse. Therefore, the computation of a bottleneck radius should always be accompanied by visual inspection when a specific target is of particular interest.

#### 3.1.1. Radius distribution and correlation with the Matthews coefficient

**Figure 6:**
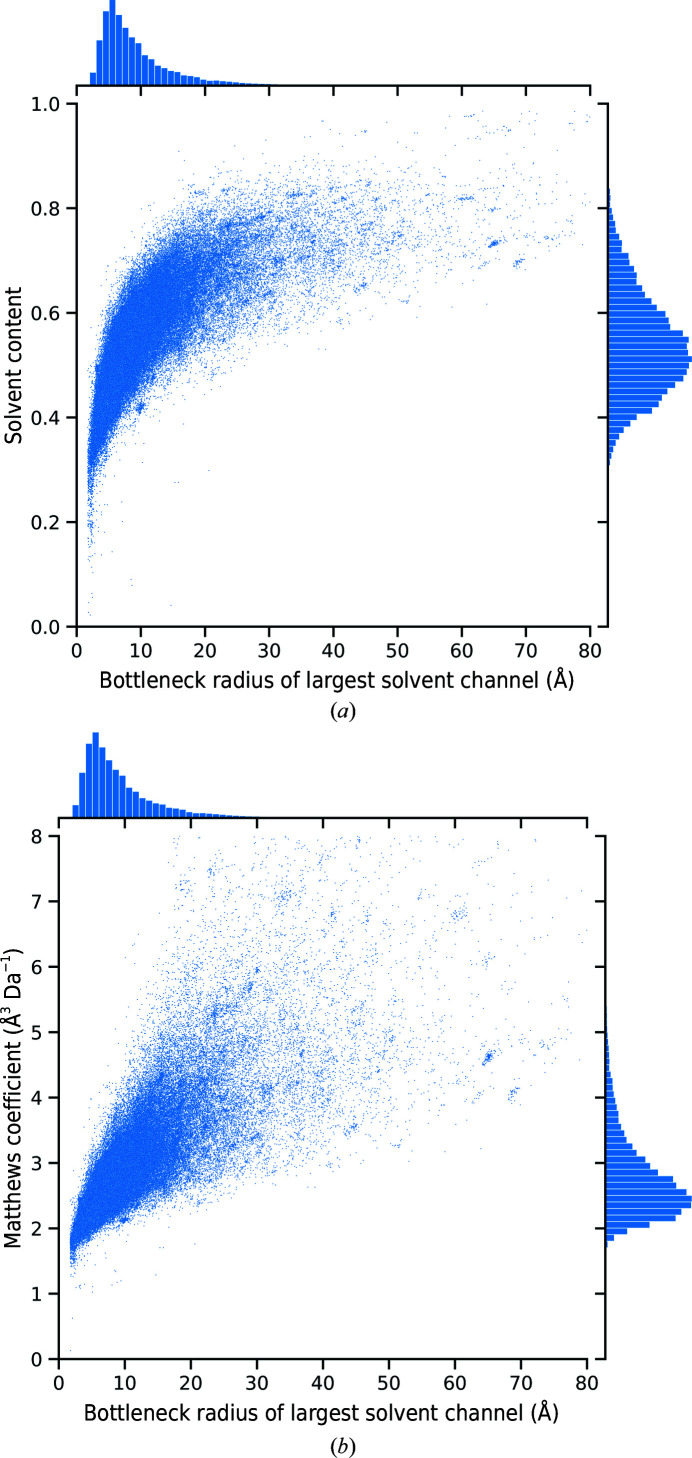
The correlation between the overall bottleneck radius and the solvent content (*a*) and the Matthews coefficient (*b*) in the form of scatter plots with univariate histograms. Outlier values with a Matthews coefficient greater than 8 Å^3^ Da^−1^ or a bottleneck radius greater than 80 Å are not displayed for improved visualization.

To estimate the benefit of predicting a solvent bottleneck radius for a user, we compared this value for every considered structure with the corresponding solvent content and Matthews coefficient (Fig. 6 ▸). Despite the inverse relation between the Matthews coefficient and the solvent content, both comparisons are instructive. The Matthews coefficient, on the one hand, has the advantage that structures with a large solvent content are better distinguished from each other since the volume per mass has no upper limit. The solvent content, on the other hand, is more intuitively interpretable.

Both parameters correlate with the bottleneck radius, illustrating that a structure with high solvent content is likelier to have large channels. In particular, large channels with bottleneck radii of more than 20 Å can almost exclusively be found in structures with greater than 50% solvent content. However, the relation is far from strict, as supported by the linear Pearson correlation coefficient of 0.61 between the bottleneck radius and the Matthews coefficient. In general, many examples of narrow bottlenecks paired with comparably large Matthews coefficients can be found. At the same time, other structures show tubular main channels in a heavily protein-occupied unit cell, leading to a low Matthews coefficient but a large bottleneck radius.

#### 3.1.2. The example of phosphoribulokinase from *Rhodobacter sphaeroides*

**Figure 7:**
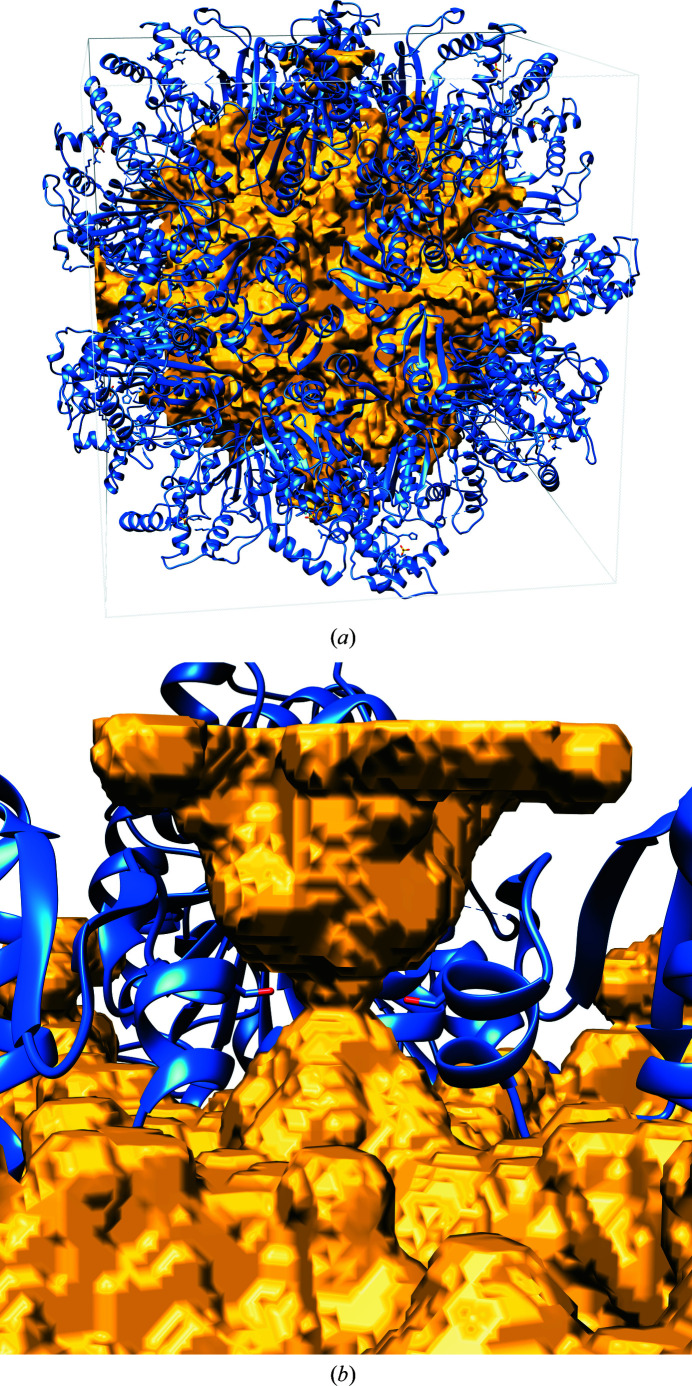
Cubic crystal of phosphoribulokinase from *R. sphaeroides* (PDB entry 1a7j) with its channel volume visualized with a cutoff radius of 2.24 Å. (*a*) The whole unit cell with the main channel. The 24 symmetric copies enclose a sphere of solvent that is only reachable by a small channel with a bottleneck radius of 2.24 Å. (*b*) The corresponding bottleneck, in which two of the four Ser248 residues are depicted. Images were created with *UCSF Chimera* (Pettersen *et al.*, 2004 ▸).

An example of a very small bottleneck radius paired with a very large solvent content was observed in the structure of phosphoribulokinase from *R. sphaeroides* crystallized in the cubic space group *P*432 (Harrison *et al.*, 1998 ▸; PDB entry 1a7j). Considering the solvent content of 57.2% and the Matthews coefficient of 2.88 Å^3^ Da^−1^, one would initially not expect an obstacle for soaking. However, the bottleneck radius of the main channel is only 2.24 Å. Since this is an extremely small radius for a crystal with such a high solvent content, we inspected the structure visually. As shown in Fig. 7 ▸, the 24 symmetry-related copies of the protein form a spherical enclosure of the solvent-occupied space in the middle of the unit cell. This space, however, is only reachable by a very narrow path from each unit-cell surface, which exhibits a bottleneck defined by four symmetric α-helices from four protein copies. The closest residue in the α-helices is Ser248.

### 3.2. Comparing channel bottleneck radii with ligand radii for a data set of soaked structures

To validate *LifeSoaks*, we assessed its ability to correctly predict whether small molecules can traverse a crystal and whether these molecules can also reach their respective binding sites. To this end, we extracted a data set of protein–ligand crystal structures obtained by soaking with small molecules from the PDB (see Section 2.2.1). The minimal projection radii of the conformers of the corresponding small molecules and ions were calculated as described in Section 2.2.2. Binding sites and their corresponding bottleneck radii were determined with *LifeSoaks*. For 33 of the 4718 binding sites, predicting binding-site-specific bottleneck radii was impossible. These are either molecules with elements that are currently not supported by the chemical model of NAOMI or molecules that are too close to a neighboring protein atom so that they are regarded as covalently bound to the protein and are discarded as ligands. The PDB codes and ligand identifiers of these complexes are listed in Supplementary Table S1.

The data set was split based on the number of conformations of the analyzed molecules. Altogether, 2230 of the 4718 molecules from the complete set are rigid and have only one conformation, with many of them being metal ions. As their calculated ligand projection radii do not depend on the predicted small-molecule conformations and therefore do not depend on the performance of the applied conformer-generation method, we first analyzed this subset.

For 100 bound ligands and ions, the binding-site accessibility could not be predicted considering both the radius in front of the pocket and that inside the pocket, hinting at extremely exposed pockets. Most of these sites are situated on the surface of the protein or in very shallow regions. Therefore, they may not include any annotated Voronoi elements, causing failure of the method. For 174 structures, a radius immediately in front of the binding site could not be predicted and the radius inside the binding site was predicted to be zero. This is caused by local bottleneck radii smaller than the applied cutoff radius, which leads to unreachable cavities. These cases usually represent fully enclosed voids inside the protein. It can be expected that most of these sites will rarely be relevant to the search for small-molecule modulators of protein function, for example, in crystallographic fragment screens. They were ignored in the following, leaving 1963 sites for more detailed analyses.

#### 3.2.1. Predicting the soakability of binding sites with single-conformer ligands and ions

**Figure 8:**
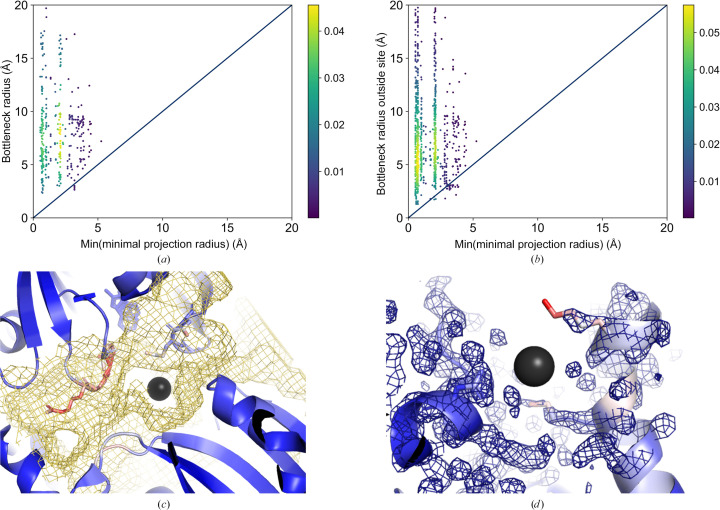
Crystal and binding-site bottleneck radii and their relation to the minimum projection radii of molecules with a single calculated conformer. (*a*) The crystal bottleneck radii are plotted against the minimum projection radius of the individual soaked molecules. (*b*) The bottleneck radii observed in front of the small-molecule-binding site are plotted against the minimum projection radius of the molecules. The line of identity is provided to highlight false-negative results. The points are colored according to the kernel-density estimate using Gaussian kernels visualizing highly occupied regions in the data set. (*c*) Loop flexibility resulting in false-negative predictions with *LifeSoaks* as shown for the structure of human oxidized purine nucleoside triphosphate hydrolase (PDB entry 6glg). The atom and cartoon representations are colored according to their *B* factor and the black sphere indicates the bottleneck position. The channels predicted by *LifeSoaks* are depicted in yellow with a cutoff radius of 2 Å. (*d*) Insufficient electron-density support for bottleneck-constituting residues leading to false-negative *LifeSoaks* predictions, as illustrated using the structure of mevalonate diphosphate decarboxylase (PDB entry 6e2v). The electron density (2*F*_o_ − *F*_c_) is shown as a grid at a sigma level of 1. Residues within a 5 Å environment of the bottleneck position (black sphere) are shown as sticks and their atoms are colored according to their *B* factor. (*c*) and (*d*) were generated with *PyMOL* (Schrödinger).

For 98.5% of the binding sites, we could correctly predict their accessibility to small molecules when only considering the minimum projection radii, with only 30 sites not predicted to be soakable. In eight cases, the main channel bottleneck radius was not predicted to be large enough to allow the ligands to traverse the crystal (Fig. 8 ▸ *a*). These eight human oxidized purine nucleoside tri­phosphate hydrolase structures were determined in complex with the fragment 1*H*-imidazo[4,5-b]pyridin-2-amine (PDB ligand ID BU8) with a minimum projection radius of 3.19 Å. According to the experimental data section of the PDB files, the fragment was soaked into the crystal. However, a publication is lacking for these examples, so we could not further assess whether this agrees with the actual experimental conditions. A closer examination of the solvent channels in the structure of this enzyme (PDB entry 6glg) shows that the bottleneck is formed by poorly resolved loop regions that might be sufficiently flexible to allow an increase in the bottleneck radius. These regions spanning residues 24–28 and 141–143 are characterized by high *B* factors, indicating a flexible region. Its residue movements might enlarge the solvent channel. Indeed, the bottleneck radius of the main channel of the crystal and that in front of the binding site increase from 2.98 Å to 4.12 and 3.95 Å, respectively, if residues with at least four atoms with an *EDIA* (Meyder *et al.*, 2017 ▸) below 0.8 are not considered in the *LifeSoaks* calculation (Fig. 8 ▸ *c*).

For the remaining 1955 structures, we analyzed the accessibility of the small-molecule-binding sites from channels in the crystal (Fig. 8 ▸ *b*). We visually inspected the structures of the 22 false-negative predictions. In general, the differences between the minimum projection radius of the ligands and the bottleneck radius of the corresponding site are very small. They can most likely be attributed to small channels leading to the binding site, which is on the edge of our simplified model of a ligand diffusing through the crystal, as passage also depends on the local radii of the ligand. The difference is greater than 0.5 Å in only eight cases, which will be discussed below.

In the structure of leukotriene A4 hydrolase with PDB code 3fuh (Davies *et al.*, 2009 ▸), the ligand with the PDB three-letter code 5H1 (1*H*-indol-5-ol, minimum projection radius of 3.69 Å) is nearly fully enclosed by the protein chain, with only a narrow channel leading to its binding site. A loop region closes over the ligand and the loop residues might be flexible, enabling the ligand to reach the binding site in a more open conformation. Moreover, the enzyme has high intrinsic flexibility and is characterized by open and closed states (Stsiapanava *et al.*, 2017 ▸). These conformational differences cannot be captured only considering the rigid crystal structure.

In another case, one sulfite ion ( 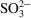 , PDB three-letter code SO3 in chain *A* with residue sequence number 537) of the cytochrome *c* nitrite reductase structure with PDB code 3lg1 (Trofimov *et al.*, 2012 ▸) is flanked by the helices of three asymmetric units and the bottleneck is formed by Val454 in chain *A*. Intriguingly, the helix ends constituting the bottleneck are characterized by high average residue *B* factors. A similar situation is observed for the structure of the same enzyme (PDB entry 2zo5; Polyakov *et al.*, 2009 ▸) and the corresponding binding site of a nitrite ion (PDB ligand code AZI with residue sequence number 530 in both chains *A* and *B*). The termini of the helices closing the binding site are characterized by high temperature factors. This flexibility might enable entrance of the ion into the binding site through a channel.

Similar observations were made for several structures of cytochrome *c* peroxidase [PDB entries 4jm8, 4jm6, 4jma, 4jmw (Rocklin *et al.*, 2013 ▸), 1aed, 1aeo, 1aeg, 1aem, 1aee, 1aen, 1aef, 1aeb (Musah *et al.*, 2002 ▸), 1aeu, 1aet and 1aes (Fitzgerald *et al.*, 1996 ▸)] with PDB ligand IDs 26D, LG3, DTI, 3FA, 2AP, IPH, 4AP, MPI, ANL, 25T, 2MZ, 3AP, 1MZ, 3MT and IMD, respectively. A loop region spanning residues 190–195 encloses the binding site of the respective ligands. This loop was reported to be highly flexible in the apo structure of the protein (Fitzgerald *et al.*, 1996 ▸). For the PDB entry 4jm8 (Rocklin *et al.*, 2013 ▸), we observe a bottleneck with a radius of 2.92 Å, which is smaller than the minimum projection radius of the ligand (3.87 Å). In addition, this bottleneck is not situated at the entrance to the corresponding site but in a channel blocked by heme. After omitting residues with low *EDIAm* values, which indicate poorly defined electron density and high flexibility, the bottleneck radius increases to 9.89 Å. Thus, the pocket is reachable by the solvent channels in the crystal. In the ligand-occupied state, this loop closes and the ligand is located in the center of a very narrow channel.

For two caesium ions (ligands with residue name CS and residue sequence numbers 502 and 501 in chains *B* and *C*, respectively) in the structure with PDB code 6hfb (Zakrzewska *et al.*, 2019 ▸), the bottleneck radii in front of the binding site were calculated to be smaller than their radii. However, the radii inside and in front of the pocket are identical, explaining this finding: these two ions lie on the protein surface and their sites are defined by only two residues, rendering the binding-site determination by *LifeSoaks* unreliable. However, we do not consider this to be a limitation of the method because users are expected to be mainly interested in druggable and sufficiently buried binding sites.

Examples of the latter four typical challenges for predicting the crystal soakability and binding-site accessibility are depicted in Supplementary Fig. S4.

The last case is the binding site of a beryllium trifluoride ion (minimum projection radius of 2.66 Å) in a structure of mevalonate diphosphate decarboxylase (PDB entry 6e2v; Chen *et al.*, 2020 ▸). The side chain of Lys67 with very high atomic *B* factors forms the bottleneck in front of the pocket (2.65 Å). Its side-chain conformation lacks proper electron-density support and might be present in various conformations in the crystal (Fig. 8 ▸ *d*).

In summary, *LifeSoaks* correctly predicted crystal soakability and binding-site accessibility for most of the true-positive examples. However, if the proteins under investigation contain highly flexible regions, the predictions should be carefully evaluated. In particular, flexible protein regions that might block the binding site in the ligand-occupied state but are more flexible in the ligand-free structure must be considered when assessing the performance of the tool. All cases in which our tool fails to correctly predict the soakability are connected to intrinsic features of the corresponding protein structure models, such as flexibility and poor electron-density support for atoms. The latter might be attributed to high flexibility or an overall poor crystal structure resolution.

#### 3.2.2. Predicting the soakability of binding sites with multiple-conformer ligands

**Figure 9:**
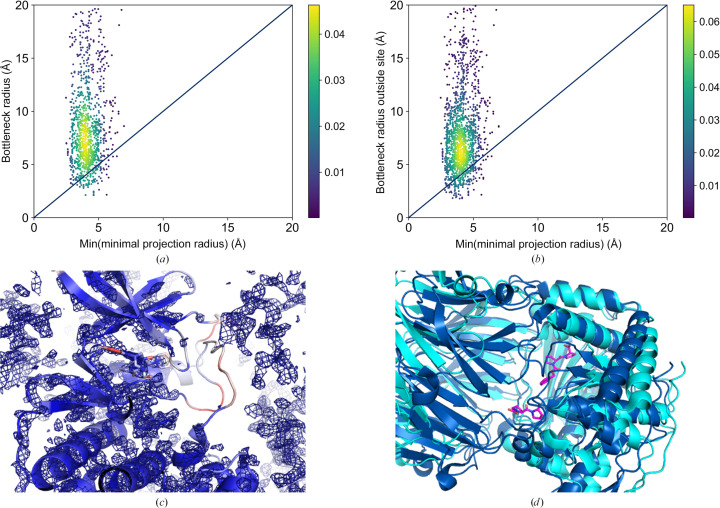
Crystal and binding-site bottleneck radii and their relation to the minimum projection radii of the conformational ensembles of the soaked small molecules. (*a*) The crystal bottleneck radii are plotted against the minimum of the minimum projection radius of the conformational ensemble of the individual soaked molecules. (*b*) The bottleneck radii observed in front of the small-molecule-binding site are plotted against the minimum of the minimum projection radius of the conformational ensemble of the molecule. The line of identity is provided to highlight false-negative results. The points are colored according to the kernel-density estimate using Gaussian kernels visualizing highly occupied regions in the data set. (*c*) The modeled structures of flexible loops that lack proper electron-density support, leading to false-negative predictions with *LifeSoaks* as shown for the structure of the human protein kinase CDK2 (PDB entry 4ez7). The atom and cartoon representations are colored according to their *EDIAm* values. The electron density (2*F*_o_ − *F*_c_) is shown in grid representation at a sigma level of 2. (*d*) Huge domain movements because of induced-fit phenomena resulting in false-negative *LifeSoaks* predictions, as illustrated by the structure of prolyl endopeptidase from *Aeromonas caviae* (PDB entry 3muo, blue). The original structure of the proteins in the crystals used for soaking highlights the accessibility of the binding site in the ligand-free crystal of the structure (PDB entry 3ium, cyan). The binding site is highlighted by the corresponding ligands in stick representation (ZPR_A_701 and ZPR_B_702, magenta). (*c*) and (*d*) were generated with *PyMOL* (Schrödinger).

A slightly different scenario was observed for larger molecules with multiple conformations (2445 ligands; Figs. 9 ▸ *a* and 9 ▸ *b*). Bottleneck radii inside and outside the pocket could not be calculated for six sites. In 46 cases, the binding-site-defining point was predicted to be inaccessible (completely enclosed by the protein). Therefore, a bottleneck outside the pocket was not calculated. For the remaining 2393 structures in this subset, the calculated bottleneck radii (crystal and binding-site bottleneck radii) are often smaller than the minimum projection radius of the conformational ensemble of the corresponding molecules compared with the set of rigid small molecules (231 false-negative predictions). For 148 of these false-negative predictions, the bottleneck radius of the crystal was predicted to be smaller than the minimum projection radius of the conformational ensemble of the soaked ligand. However, most data-set structures were correctly predicted as soakable (90.1%).

The higher number of structures whose soakability was not correctly predicted may be attributed to the molecules being larger than those in the first data set. This finding highlights the boundaries of the basic assumption that we can predict soakability by approximating the minimum dimensions of a molecule by a 2D-projected circle of minimum radius and annotating channels by spheres. Additionally, inaccuracies in the conformational sampling, especially in the case of large molecules with many rotatable single bonds, might occur. Finally, some of the proteins whose flexibility was discussed earlier can also be found in this data set, only with different ligands (for example human oxidized purine nucleoside triphosphate hydrolase structures, PDB entries 6gle, 6gli, 6gln, 6glr, 6gls and 6glv). Others are new challenges for soaking prediction that can only be faced by careful visual inspection of the protein structures under investigation. To this end, we sorted the results according to the difference between the bottleneck and the minimum projection radii in descending order. An analysis was performed starting with the prediction with the highest difference between the minimal projection radius and the predicted bottleneck radius. We will discuss some examples with especially large differences.

The example with the highest difference between the bottleneck and the minimum projection radius is the protein kinase structure with PDB code 4ez7 (Martin *et al.*, 2012 ▸). Both the crystal bottleneck radius and the bottleneck radius of the channel leading to the binding site for 8-anilinonaphthalene-1-sulfonic acid (residue 2AN with residue sequence numbers 302 and 303 in chain *A*) and staurosporine (residue STU with residue sequence number 301 in chain *A*) are smaller (2.16 Å) than the minimum projection radii of the soaked molecules (4.62 and 5.63 Å, respectively). However, large regions of the modeled structures are not properly supported by electron density due to low resolution and high flexibility (Fig. 9 ▸ *c*). The arrangement of the asymmetric units in the unit cell might also allow loop flexibility. The ligand is flanked by loop regions with high temperature factors and no reliable electron density can be found for the modeled activation loop of the kinase. Upon removing un­reliably modeled residues according to their *EDIA* value (at least four atoms with *EDIA* < 0.8), the bottleneck radius of the structure increases to 6.55 Å, which can be attributed to the omitted loop regions opening a broader channel through the crystal (Fig. 9 ▸ *c*). These results indicate that low electron-density support causes reduced prediction reliability. The same holds for the protein kinase structures with PDB codes 3pxz, 3py1 and 3pxq (Betzi *et al.*, 2011 ▸).

Another interesting example is the structure of (*S*)-1-phenylethanol dehydrogenase (PDB entry 2ewm; Höffken *et al.*, 2006 ▸). It has a very small crystal bottleneck radius of 2.63 Å. Intriguingly, a structural region spanning residues 188–206 of chain *B* is characterized by very high *B* factors. Unfortunately, an electron-density map is not available from the PDBe (Armstrong *et al.*, 2020 ▸) to check the validity of the modeled atom coordinates. However, a ligand-free structure of this enzyme (PDB entry 2ew8) crystallized in a different space group and is characterized by different unit-cell parameters. In this structure, residues 188–206 are not resolved, leading to a much higher calculated crystal bottleneck radius of 9.5 Å. According to the authors of the structure (Höffken *et al.*, 2006 ▸), the crystal used to obtain the NAD-bound structure was grown under the same conditions. It broke apart upon soaking and one piece was immediately cooled for structure determination. Therefore, it cannot be excluded that the ligand traversed the crystal in the apo form with the former space group, giving rise to changes in the crystal after soaking. Upon omitting the discussed flexible regions from the *LifeSoaks* calculations, the calculated bottleneck radius increases to 4.0 Å, which is still smaller than the minimum projection radius of the soaked NAD molecule (5.04 Å). Omitting another region that was not resolved in the ligand-free structure and was characterized by high *B* factors in the soaked structure (residues 188–192 of chain *A*) leads to a bottleneck radius of 4.4 Å that is at least similar to the predicted minimum projection radius of NAD.

The structure of prolyl endopeptidase (PDB entry 3muo) exhibits a large crystal channel of 22.8 Å. Nevertheless, its binding sites are inaccessible due to high enclosure by the protein. Thus, there must be another reason for its soakability than local protein flexibility. In the crystallized opened state of the enzyme (for example PDB entry 3ium; Li *et al.*, 2010 ▸) its two domains are apart from each other, leading to highly accessible binding sites (Fig. 9 ▸ *d*). These open-state crystals were used to soak ligands into the structure (Li *et al.*, 2010 ▸), although the soaked structure is characterized by different crystal properties. Indeed, the authors of the structure explained this observation. They argue that due to the high solvent content of the crystal (62%), huge conformational changes of the enzyme are well tolerated. They do not interfere with crystal integrity due to the loose packing. These differences explain why *LifeSoaks* could not correctly predict the soakability of the crystal in the ligand-bound form. Therefore, it is advisable to exploit structures of the native crystal to be soaked for predictions with *LifeSoaks*. The same holds for the structure of this enzyme for PDB entry 3ivm (Li *et al.*, 2010 ▸; 57% solvent content).

Similarly, we find a large solvent channel that traverses the crystal in the structure of aminopeptidase N with PDB code 2dqm (Ito *et al.*, 2006 ▸). However, the binding site of the soaked small molecule bestatin is connected to this channel by a narrow channel with a bottleneck radius of only 1.8 Å, which is much smaller than the minimum projection radius of the soaked molecule (3.95 Å, residue name BES). The bottleneck is formed by Lys852 and Arg845 from two neighboring asymmetric units. These residues are characterized by higher *B* factors than the average for the structure and might be sufficiently flexible to allow ligands to traverse. Considering this flexibility and the high solvent content of 66%, it might be possible that conformational changes accompany the binding of the ligand. Different conformational states of aminopeptidase N are also discussed in the literature (Addlagatta *et al.*, 2006 ▸).

The structure of lysozyme in complex with the soaked dye bromophenol blue (PDB entries 6syc, 6syd and 6sye, tetragonal space group; Plaza-Garrido *et al.*, 2020 ▸) also represents an interesting explanation for false-negative predictions regarding the ability of small molecules to traverse a crystal. The crystal bottleneck radii are considerably smaller than the minimum projection radius of the dye (5.4, 4.8 and 3.8 Å, respectively, versus 5.8 Å). The bottleneck radii in front of the binding site are even smaller, contradicting the explanation in the corresponding publication (Plaza-Garrido *et al.*, 2020 ▸). Its authors used *MAP_CHANNELS* and the radius of gyration of the dye (4.6 Å), which underestimates the molecular dimensions, to analyze the accessibility of two sites in the crystal. However, our calculations cannot confirm that the soakability can be explained without considering the flexibility of the protein. Intriguingly, a larger bottleneck radius of 5.4 Å was found for the structure in an orthorhombic space group (PDB entry 6syc). For the corresponding ligand-free orthorhombic (PDB entry 6f1o) and tetragonal (PDB entry 6f1p) lysozyme crystals (Plaza-Garrido *et al.*, 2018 ▸), similar bottleneck radii were predicted. As the bottleneck is lined by less flexible proline and serine residues (Pro79 and Ser86), there might be another explanation for the soakability of this dye molecule. The nonspherical 3D conformation of the soaked molecule stands out. The assumption that a single minimum projection radius describes a molecular conformation is too simple to explain the soakability of the crystal. An ellipsoid describes the form of the molecule much better and might lead to better predictions. Also, different conformations of the molecule might play a crucial role for a ligand traversing a crystal. Although we analyzed the minimum projection radii of the conformers to obtain the smallest one, we cannot exclude that a more comprehensive conformer sampling might lead to conformers with even smaller minimal projection radii. Also, local minimal projection radii for various conformations might play a role in crystal traversal of small non-globular-shaped molecules.

In summary, the reasons why *LifeSoaks* might fail to predict that crystals can be soaked with ligands are high local flexibility, domain movements, missing electron-density support for modeled structures, a missing ability to cope with PDB structures containing multiple models, an insufficient sampling of the small-molecule conformations leading to inaccurately predicted projection radii, an inadequate representation of the solvent space and ligands by spheres, and the inability to detect completely enclosed sites, for example due to induced-fit phenomena. With regard to the former issues, users might modify the corresponding structures accordingly by excluding highly flexible residues (based on the *B*-factor distribution or *EDIA* calculations) to exclude unreliably modeled regions based on the electron density. Furthermore, the use of structures of the protein in the ligand-free crystals that are to be soaked is advisable. Also, users should carefully inspect the predicted channels and visualize the bottleneck positions to assess the true soakability of their crystals manually. The remaining issues cannot be fully solved without substantial further developments. However, handling protein flexibility is one of the most important issues to ensure reliable predictions in most analyzed examples.

#### 3.2.3. Comparing *LifeSoaks* and *MAP_CHANNELS*

**Figure 10:**
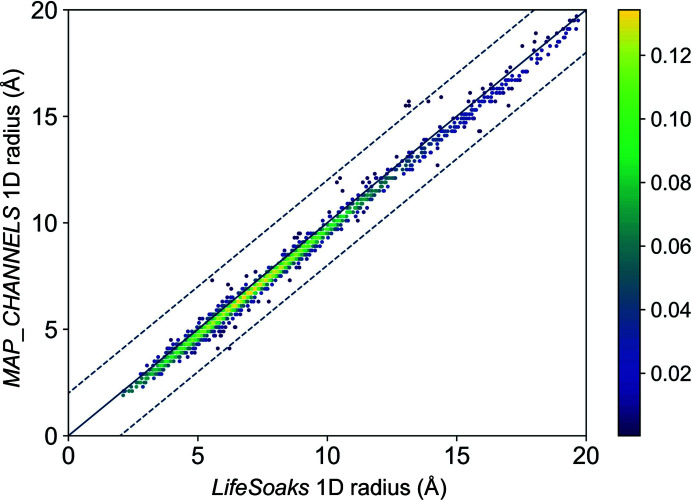
Comparison of the 1D bottleneck radii calculated by *LifeSoaks* and *MAP_CHANNELS* (1713 structures). The plot covers most data points up to 20 Å. The blue dashed lines indicate a prediction difference of at most 2 Å.

To the best of our knowledge, *MAP_CHANNELS* (Juers & Ruffin, 2014 ▸) is the only other available software for automated bottleneck calculations and soakability predictions. Although analyses of binding-site accessibilities are not feasible with *MAP_CHANNELS*, we decided to compare both tools with regard to the calculation of crystal bottleneck radii for the PDB structures in the data set. Fig. 10 ▸ shows the crystal bottleneck radii of the set of small-molecule-soaked crystal structures as predicted by *LifeSoaks* and *MAP_CHANNELS*. Major differences can only be observed in the 2D and 3D radii of the structures (Supplementary Fig. S5). The 2D and 3D radii describe the largest radius that a sphere may have to move infinitely along the 2D and 3D directions, respectively, in the crystal. They are smaller than or equal to the overall bottleneck radius, which can also be called the 1D radius. With regard to the 1D radii (the bottleneck for traversing the crystal in one direction), which are the most informative radii for a user investigating the general soakability of a crystal, both tools predict very similar bottleneck radii. In nine cases, the bottleneck radii are predicted to be more than 2 Å smaller by *LifeSoaks*. However, these cases represent crystals with very large bottleneck radii of 13 Å and above. In only one case, *MAP_CHANNELS* predicts a considerably smaller bottleneck radius (>2 Å difference). This is the structure of decay-accelerating factor (PDB entry 1ok9; Lukacik *et al.*, 2004 ▸). The outstanding feature of this structure is the expansion of the asymmetric unit through multiple unit cells, which might lead to inaccuracies in the channel analyses with *LifeSoaks*, leading to a difference of 2.1 Å.

In general, *MAP_CHANNELS* analyses lead to lower bottleneck radii compared with *LifeSoaks*, within the limitations of the chosen grid spacing for *MAP_CHANNELS* calculations. The resulting maximal radius underestimation is half of the maximal distance that any point in space can have to its closest grid point. This extreme case would be equivalent to a point exactly in the center of an eight grid-point cube, resulting in an error of 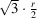 ( 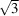 Å for our analysis). Another intriguing difference between the methods is the treatment of protein modifications, as exemplified by the predicted bottleneck radii for the structure of chlorperoxidase from *Leptoxy­phium fumago* (PDB entry 2ciz). The protein has several glycosylation sites, leading to a bottleneck radius of 10.5 Å as predicted by *LifeSoaks*. In contrast, *MAP_CHANNELS* predicted a radius of 12.1 Å, neglecting the glycosylation of the protein. After re-performing the calculation enabling the option to include nonwater heteroatoms in the distance-map calculation, the method reports a similar radius of 10.6 Å. However, a user would expect that the calculation would consider all covalent protein modifications as part of the protein. Additionally, explicitly using all heteroatoms apart from those of water molecules might lead to problems due to considering additional buffer components that can freely diffuse in the crystal in the calculation.

For the complete data set, the *MAP_CHANNELS* crystal bottleneck radii of 128 structures suggest nonsoakability of the crystals, compared with *LifeSoaks*, which predicted only 103 structures to be nonsoakable based on the crystal bottleneck radii. Notably, the accuracy of *MAP_CHANNELS* can be increased with a change in grid spacing at the expense of a longer computing time.

**Figure 11:**
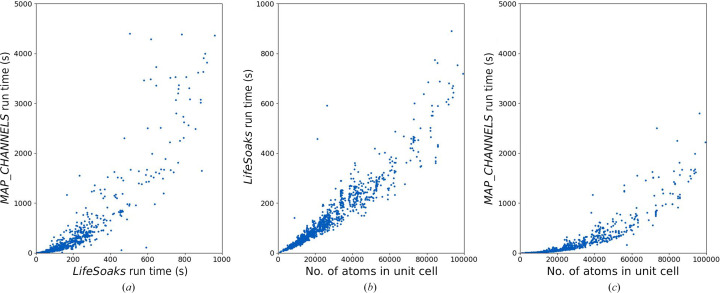
Comparison of the run times of *LifeSoaks* and *MAP_CHANNELS*. (*a*) Comparison of the run times of both tools. (*b*) Dependence of the run time on the number of atoms in the unit cell for *LifeSoaks*. (*c*) Dependence of the run time on the number of atoms in the unit cell for *MAP_CHANNELS*.

Regarding run time, the most significant differences can be found for structures with large unit cells, and approximately corresponds to the number of atoms in the system (Fig. 11 ▸). Considering all structures of the benchmark data set, both tools are similarly fast on average, 

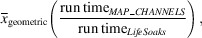

with *LifeSoaks* being 1.36 times faster than *MAP_CHANNELS*. For 1281 structures, *MAP_CHANNELS* is faster than *LifeSoaks*. However, the run-time difference exceeds 60 s in only 46 cases. The better average run time of *LifeSoaks* can be attributed to crystals with unit cells with larger numbers of atoms. When only considering systems with 50 000 and more atoms in the unit cell (12% of the data set), *LifeSoaks* is on average nearly 2.7 times faster than *MAP_CHANNELS*. This trend can be explained by the run time increasing roughly linearly [expected 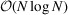 ] with the number of atoms with *LifeSoaks*, while it increases quadratically with *MAP_CHANNELS*. The latter has already been discussed in Section 1, outlining this major limitation of grid-based approaches.

Whereas the *MAP_CHANNELS* approach can be beneficial for coarse-grained analyses of solvent channels to obtain a crude estimate of the bottleneck radii, using *LifeSoaks* is preferable over using *MAP_CHANNELS* for the application scenario of predicting the bottleneck radii of crystals with small molecules to assess their applicability to soaking-based screening studies. Especially for particularly large unit cells, a concise assessment of the bottleneck radii is much faster than an analysis with *MAP_CHANNELS*. The availability of *LifeSoaks* on the Proteins*Plus* web server enables on-the-fly soaking predictions without the hurdles of installation and parametrization. In addition, it reports the positions of bottlenecks as Cartesian coordinates, facilitating the detection of soaking obstacles for users. Finally, *LifeSoaks* offers automated analyses of binding-site accessibility, a feature that is not implemented in *MAP_CHANNELS*.

### 3.3. GC11 xylanase from *Nectria haematococca*, a nonsoakable structure

Xylanases are enzymes that catalyze the degradation of the hemicellulose polysaccharide xylan and are therefore of biotechnological interest. In recently published work, the GC11 xylanase from *Nectria haematococca* (NhGC11) has been investigated with regard to substrate binding and known inhibitors (Andaleeb, 2021 ▸). However, for all compounds, soaking a monoclinic crystal of NhGC11 (Andaleeb *et al.*, 2020 ▸) did not lead to electron density, hinting at the presence of a ligand in its known binding site. The author attributed this to the dense packing of the monoclinic crystal structure with a low Matthews coefficient of 1.9 Å^3^ Da^−1^ and narrow solvent channels. This structure was compared with a related xylanase from *Trichoderma reesei*, which crystallizes in an ortho­rhombic form with a Matthews coefficient of 2.3 Å^3^ Da^−1^ and which exhibits larger solvent channels. For this xylanase, crystal structures with large substrates have successfully been obtained by soaking (Wan *et al.*, 2014 ▸).

Since the author did not use a specialized tool to analyze the solvent channels, these claims are based on visual inspection of the unit cell. Therefore, we analyzed the monoclinic NhGC11 structure with *LifeSoaks*. The structure exhibits a narrow bottleneck of the main channel with a radius of 3.83 Å, while the orthorhombic crystal contains a large solvent channel with a bottleneck radius of 9.75 Å. We believe that this narrow bottleneck is size-exclusionary for polyxyloses and potential inhibitors and thereby hinders the soaking process.

**Figure 12:**
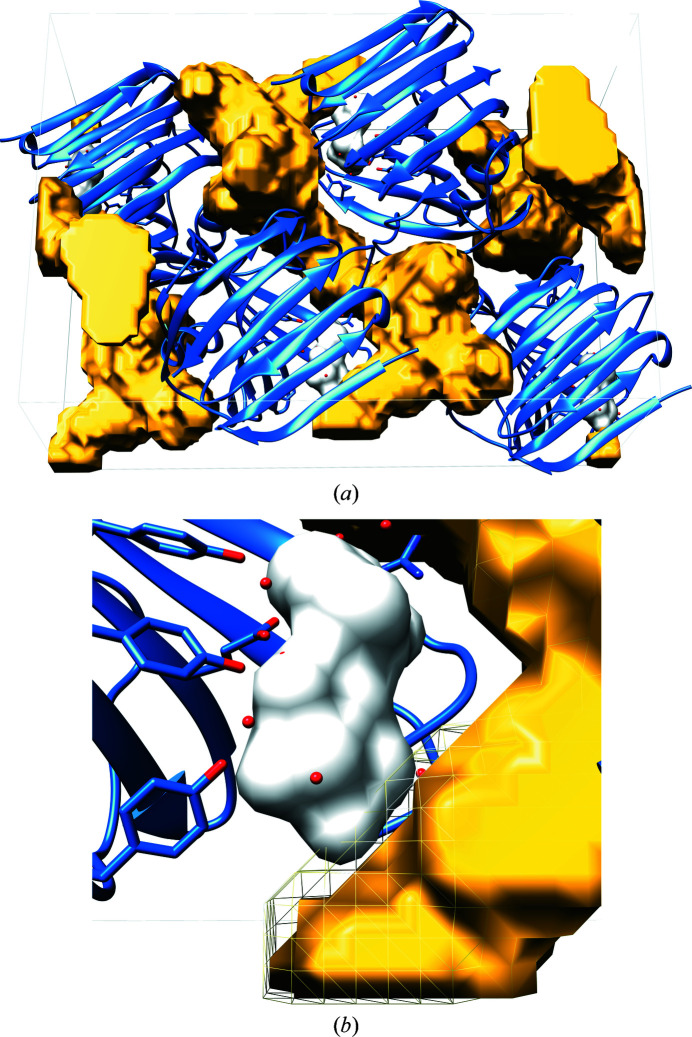
A soaking obstacle for substrate-bound xylanase in a monoclinic crystal. (*a*) One unit cell of a monoclinic NhCG11 crystal. The narrow main channel is shown in yellow, while proteins are colored blue. The xylobiose molecule is shown in white. The channel closely follows the binding-site contour. (*b*) The bottleneck position of the channel. Xylobiose in the binding pocket further narrows the radius of the channel. The channel of the empty structure is shown as a meshed grid visualizing the difference in the bottleneck size compared with the xylobiose-bound case. Images were created with *UCSF Chimera* (Pettersen *et al.*, 2004 ▸).

Furthermore, the NhGC11 channel largely follows the xylanase-binding site (Fig. 12 ▸ *a*). Xylanase is a polymer and the binding site is almost tubular. Therefore, we investigated the influence of a bound ligand on the bottleneck radius since it seems possible that a bound ligand further narrows the solvent channel, making it even harder to penetrate the crystal. Since no ligand-bound structure of NhGC11 has been published, we used a related *Bacillus subtilis* xylanase with the same fold, including xylobiose and xylose as cleavage products of xylotriose (Satyanarayana *et al.*, 2013 ▸). We aligned both xylanase structures and added the xylobiose molecule to the NhGC11 structure since this substrate reportedly could not be soaked into the crystal (Andaleeb, 2021 ▸). As expected, the inclusion of xylobiose in the Voronoi graph leads to a smaller main channel. Its radius was reduced from 3.83 to 3.54 Å (Fig 12 ▸ *b*). This further supports the hypothesis that soaking was unsuccessful due to the narrow solvent channel.

## 4. Conclusions

In this work, we present *LifeSoaks*, a novel Voronoi diagram-based tool for analyzing and visualizing solvent channels in protein crystals. While considering the periodic boundary conditions of the crystal, *LifeSoaks* determines reachability in a set-based approach that allows local storage of the bottleneck radii at each Voronoi element. Thereby, information on positions of interest can be obtained by a trivial lookup, while reachability information on larger areas can be dynamically visualized from the output CCP4 file by any common molecular viewer.

We computed the bottleneck radii for the main channels of all crystal structures in the PDB, finding that a significant number of them exhibit narrow bottlenecks that may influence the ability of large molecules to diffuse freely inside the crystal. Completely blocked channels, on the other hand, rarely appear and are usually found in small polypeptides. A comparison of bottleneck radii with Matthews coefficients revealed a weak correlation. However, it can be seen that some structures with comparably high Matthews coefficients may exhibit extremely narrow bottlenecks. Correspondingly, structures with low solvent content might still exhibit large bottleneck radii, allowing successful soaking.

To our knowledge, *LifeSoaks* represents the first grid-independent approach for crystal solvent-channel computation. A comparison with the grid-based channel-detection tool *MAP_CHANNELS* revealed that both tools find similar bottleneck radii, while *LifeSoaks* eliminates the systematic radius underestimation of a grid-based approach. At the same time, *LifeSoaks* shows a more favorable run time for an increased number of unit-cell atoms.

The implemented methodology results in a data structure where reachability information is stored locally. Besides the solvent-channel visualization and overall bottleneck radius determination, this enables automatic detection of the location of the bottleneck. Furthermore, local bottlenecks for sites can be computed, which is especially important in small-molecule soaking. This feature improves the interpretability of the results, while the incorporation of *LifeSoaks* into the Proteins*Plus* web server enhances its accessibility. A command-line tool for automated calculations on multiple structures is also provided.

A limiting factor for the benchmarking was the absence of published cases of failed soaking attempts for known ligands. As a consequence, only a single example could be analyzed in detail. As an alternative, we created a data set of protein–ligand complexes in the PDB generated by soaking according to the authors of the structures. We found that the majority of computed bottleneck radii are in agreement with the experimental results. The observed false-negative results were usually a result of flexible protein regions or poor electron-density support. A rigid model cannot properly represent these cases, and when in doubt the computed channels should be visually inspected by the experimenter, considering all additional information that an expert has on the crystal of interest. When detailed knowledge about crystal flexibility exists, channel calculations may be repeated for several conformations to investigate the behavior of the channel.

Overall, we believe that *LifeSoaks* channel calculations preceding soaking experiments can be helpful for anticipating problematic areas, and we suggest that crystallographers routinely apply them in their preparation. Finally, the mere visualization of solvent-filled areas might be valuable in any other context where intra-crystalline dynamics are of interest.

## 5. Related literature

The following references are cited in the supporting information for this article: Attali & Boissonnat (2003 ▸), Dwyer (1989 ▸) and Erickson (2001 ▸). 

## Supplementary Material

Supporting Information including Supplementary Figures and Tables. DOI: 10.1107/S205979832300582X/nz5014sup1.pdf
