# Supplementary material for: LifeSoaks: a tool for analyzing solvent channels in protein crystals and obstacles for soaking experiments
Source: Acta Crystallogr D Struct Biol. 2023 Aug 10;79(Pt 9):837–56. doi: 10.1107/S205979832300582X (PMC10478636; doi:10.1107/S205979832300582X)
Supplement: Supplementary file 1 [file d-79-00837-sup1.pdf]

STRUCTURAL
BIOLOGY

Volume 79 (2023)

Supporting information for article:

LifeSoaks: a tool for analyzing solvent channels in protein crystals and obstacles for soaking experiments

Jonathan Pletzer-Zelgert, Christiane Ehrt, Inken Fender, Axel Griewel, Florian Flachsenberg, Gerhard Klebe and Matthias Rarey

S1 Data

Data sets discussed in this paper are provided at the rareylab GitHub account

(https://github.com/rareylab/LifeSoaksPaper_Data).

S2 Run time

As described in Section 3.1, a bottleneck calculation was performed for 167,408 structures from the PDB. For these computations, we measured the run time (Figure S1), which resulted in a mean of 147.1 seconds and a median of 61.0 seconds. 161,806 structures (96.7%) could be processed in less than 10 minutes and 166,501 (99.5%) in less than 30 minutes, while 907 exceeded this limit. When visualizing the run time in relation to the number of heavy atoms per unit cell (Figure S2), it can be seen that most of these cases represent large structures with more than 200,000 heavy atoms per unit cell.

An increasing run time with increasing atom count is expected for an algorithm that performs in expected $\mathcal{O}(N \cdot log(N))$ time. However, in a small number of cases, the run time seems to grow faster, which is in agreement with the theoretical worst-case $\mathcal{O}(N^2)$ performance of the algorithm. In total, 47 cases show a run time of more than three hours even though they all have less than 200,000 heavy atoms in the unit cell. This behavior might be caused by a suboptimal atom distribution, such as co-planar atom positions leading to a worst-case behavior. However, an investigation of the underlying geometric causes is beyond the scope of this work and we refer the reader to the extensive literature on the worst-case and average-case complexity of Delaunay tetrahedralizations (Amenta *et al.*, 2007; Attali & Boissonnat, 2003; Dwyer, 1989; Erickson, 2001).

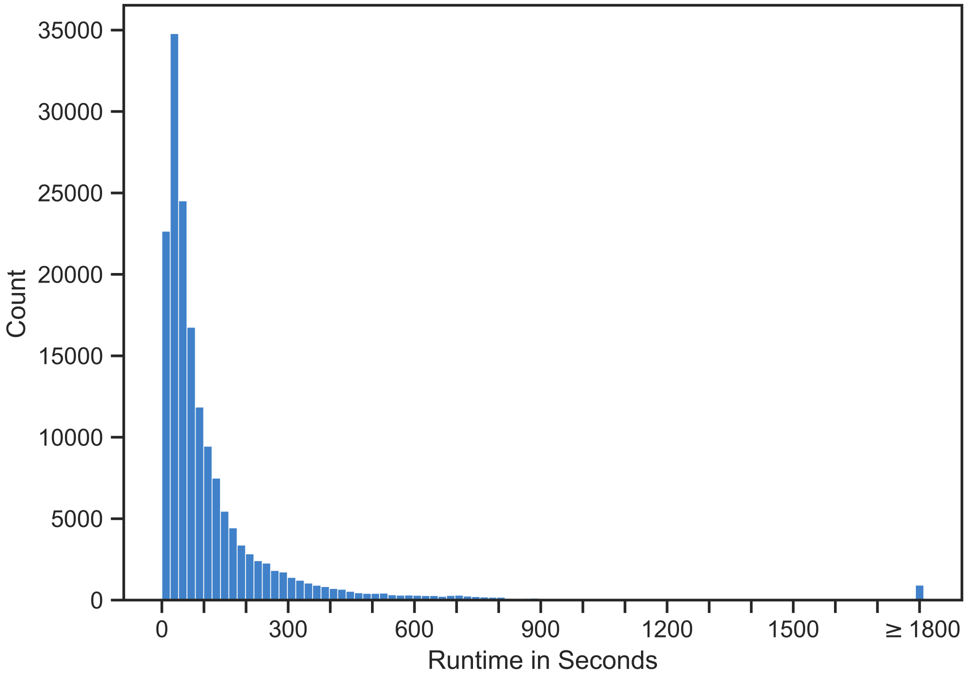

Fig. S1. Histogram of run times for calculations on 167,408 successfully processed PDB files. 91 bins (20 seconds each) for up to 30 minutes (1800 seconds) run time are displayed. The last bin includes all run times larger than or equal to 1800 seconds

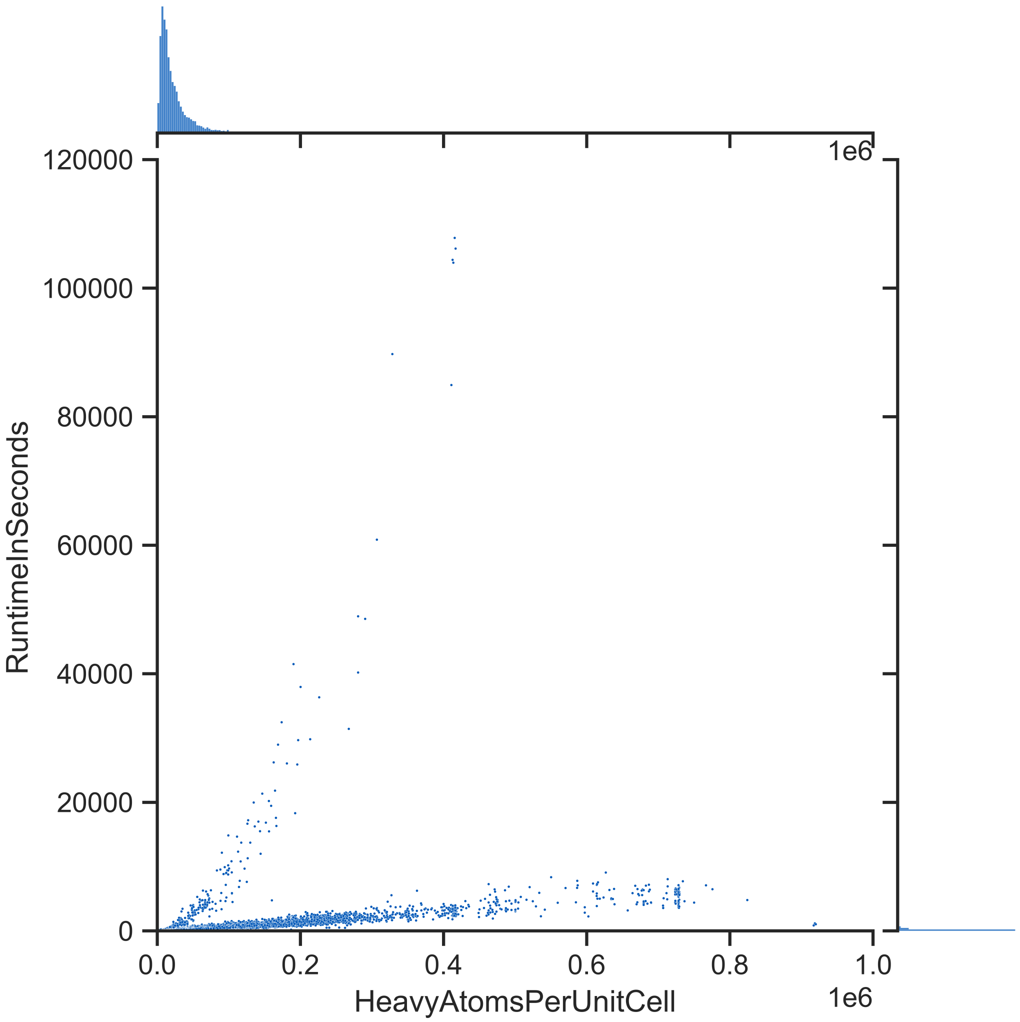

Fig. S2. Scatter plot visualizing the run time in relation to the number of heavy atoms in a unit cell. While most cases are in agreement with an expected $\mathcal{O}(N \cdot log(N))$ run time, a small number of cases seems to follow the worst-case run time behavior of $\mathcal{O}(N^2)$.

S3 Space to consider for constructing a correct unit cell Voronoi diagram

In order to construct a correct Voronoi diagram of a complete unit cell, its periodic condition needs to be considered. To this end, some of the surrounding space needs to be considered, since neighboring atoms may influence distant Voronoi elements. Since the periodic input space is theoretically infinite, we determine a suitable subspace of the input that guarantees a correct result while minimizing the size of the input.

Let $\bar{U}$ be the space of the central unit cell and P be the infinite periodic set of atom coordinates in the crystal for which an infinite Voronoi diagram V exists. Our goal is to compute $V_{\bar{U}} = V \cap \bar{U}$.

We consider an arbitrary position $\bar{s} \in \bar{U}$. In general, depending on the number of equally distant closest points in P, $\bar{s}$ may be part of a Voronoi vertex (four points), edge (three points), facet (two points) or polyhedron (one point) in $V_{\bar{U}}$.

Without loss of generality, we consider a single point $\bar{p} \in P$ that is one of the closest points to $\bar{s}$. Now we consider the set S that includes $\bar{s}$ and all its periodic copies. We first notice that $\forall_{s \in S} : ||\bar{p} - \bar{s}|| \leq ||\bar{p} - s||$.

If an $s \in S$ exists that is closer to $\bar{p}$ than $\bar{s}$, a periodic copy of $\bar{p}$ exists that has the same smaller distance to $\bar{s}$. However, that is a contradiction to the fact that $\bar{p}$ was one of the closest points to $\bar{s}$.

From this distance restriction, we limit the space that can contain $\bar{p}$ to the subspace that is closest to $\bar{s}$ among S. From the definition of a Voronoi diagram follows, that this space is a Voronoi polyhedron $H(\bar{s})$ around $\bar{s}$ on S which can be efficiently computed.

From this, we can derive the subspace $H(\bar{U}) = \bigcup_{s \in \bar{U}} H(s)$ as well as $P_{\bar{U}} = P \cap H(\bar{U})$. By using $P_{\bar{U}}$ as input we guarantee that all points in P that are closest to at least one position in $\bar{U}$ are considered. We thereby can correctly calculate $V_{\bar{U}}$.

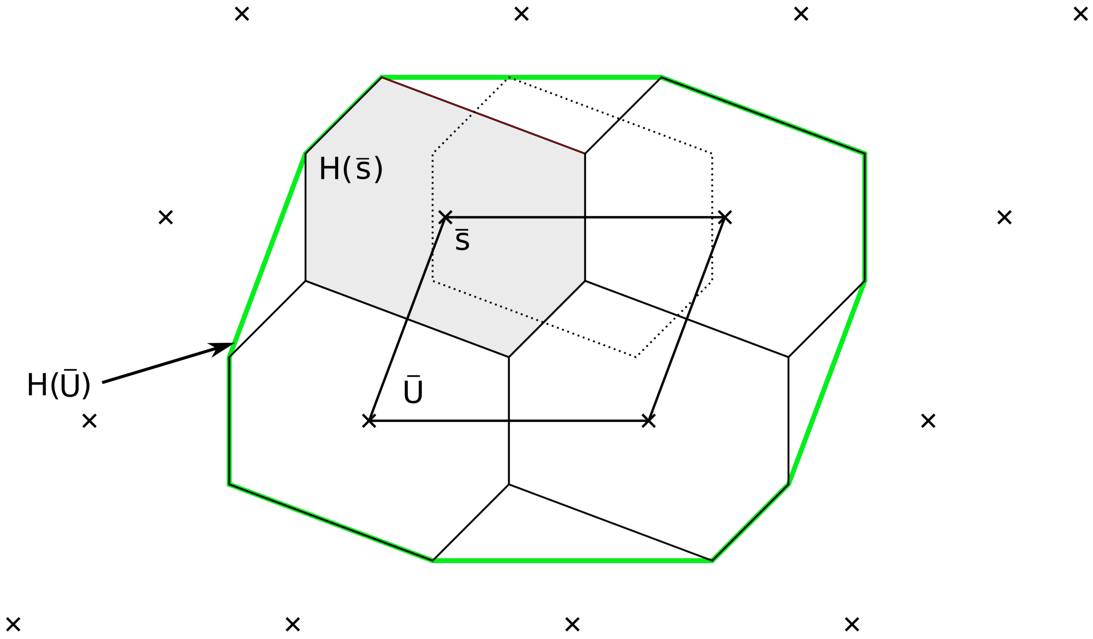

Fig. S3 A 2D example visualizing the construction of $H(\bar{U})$. The central rectangle represents $\bar{U}$ while the cross in the upper left corner represents $\bar{s}$. All other crosses represent its periodic copies S. The grey polygon around $\bar{s}$ represents $H(\bar{s})$. The dashed-line polygon represents $H(s)$ for an arbitrary point between the upper left and upper right corner of $\bar{U}$ while the other three polygons represent H for the other corners. The green polygon represents $H(\bar{U})$ which is the convex hull of the four corner polygons or the union of all H of points in $\bar{U}$.

The remaining task is to calculate $H(\bar{U})$. Since the geometry of the unit cell exactly defines the translation of periodical copies of any point, any $H(s)$ will have the exact same shape, but will be translated depending on the position of s in $\bar{U}$. Therefore, it is sufficient to consider the border of $\bar{U}$ and move a precomputed H along these borders. We achieve this by calculating the polyhedron for each corner of the unit cell and calculating their convex hull. The principle is visualized in Figure S3.

S4 **Output**

Here the most important output statements of the command line tool are explained in detail.+

WARNING: Residue <RES_ID> is incomplete:

A residue is not fully resolved. This is relevant since the missing atoms might block a channel without *LifeSoaks* detecting it.

WARNING: Residue <RES_ID> Is on surface but has missing atoms:

The residue has missing atoms and is also located on the molecule surface. This makes it even likelier that these unresolved atoms might block a channel. A visual inspection is recommended.

Treating <MOL_ID> as molecule name:

Specifier is treated as molecule name.

Treating <MOL_FILE> as file name:

Specifier is treated as molecule file. The molecule is constructed from the additionally provided file

Ligands present in file:

U94_A_201

...:

Writes all IDs of ligands in the file. Only displayed in verbose mode

Unit cell Voronoi building started:

Reading of input files is finished. Now starts the actual Voronoi calculation which is the most demanding computation with respect to time and memory. The construction can take several minutes.

<Number> atoms in initial supercell have invalid coordinates
note that a supercell consists of 27 unit cells:

Gives the number of atoms as derived from the PDB file's SEQRES record in the initial supercell that have invalid coordinates. A supercell consists of 3x3x3 unit cells. The input space is subsequently reduced.

Converting to input took <Number> seconds:

This is the time it took to convert the atoms into Voronoi input. This step includes the restriction of the input space

<Number> input points used:

This is the number of points that are actually used for the computation. The input space is already restricted. Only atoms on the surface are included.

Calculating Delaunay tetrahedralization took <Number> seconds:

This is the time it took to calculate the Delaunay tetrahedralization which is a dual to the Voronoi diagram. This is the most time and memory-consuming step of the algorithm.

Converting Delaunay tetrahedralization to Voronoi diagram took <Number> seconds:

This is the time it took to convert the Delaunay tetrahedralization into a unit cell Voronoi channel graph. This includes the conversion into Voronoi vertices and edges and the cutting at the unit cell borders.

Voronoi construction took <Number> seconds in total:

This is the summed up time of the Voronoi construction steps.

Total calculation took <Number> seconds:

This is the total time the Voronoi construction and channel analysis took.

Each complex contains <Number> residue atoms:

This is the number of heavy atoms of one protein/nucleic acid complex.

<Number> of them have coordinates:

This is the number of protein/nucleic acid heavy atoms that have coordinates

Each unit cell contains <Number> heavy atoms:

This is the number of heavy atoms in each unit cell.

Solvent content: <Number>%

The solvent content of the crystal

Matthews coefficient: <Number>

The Matthews coefficient of the crystal in $\frac{\text{Å}^3}{Da}$

The bottleneck radius for the <x,y,z> dimension is: <Number> Angstrom:

The bottleneck radius of each dimension. This is the radius of the largest sphere that can pass the crystal in the corresponding direction.

The overall bottleneck radius of the largest channel is: <Number> :

The overall bottleneck radius of the largest channel. This value is the maximum of the bottlenecks in the <x,y, and z> directions.

<Number> bottlenecks were detected:

The number of main bottlenecks that where detected. These bottlenecks are only the ones of the largest main channel and should all have the same radius, but since crystals often have numerous non-crystallographic symmetries, these bottlenecks can exist multiple times.

Position of bottleneck: <Coordinate>

Fractional position of bottleneck: <Fractional_Coordinates>

Closest residue(s): <RES_ID>

...:

The position of this bottleneck. It is given in absolute and fractional coordinates. Furthermore, the closest residues are given.

The following bottleneck radii have been found for binding sites:

For binding site defined by: <REF_LIG or FILE>

Position of binding site center: <Coordinates>

Bottleneck radius inside binding site: <Number>

Bottleneck radius in front of binding site: <Number>:

If a binding site is specified, gives the coordinates of the site center. Furthermore the bottleneck radii of the inside and in front of the binding site are given.

Position of bottleneck: <Coordinates>

Fractional position of bottleneck: <Fractional_Coordinates>

Closest residue(s): <RES_ID>

...: Below the binding site information, the location of the corresponding bottleneck is given. This is analogous to the main bottleneck position.

S5 Binding site bottleneck radius annotation failures

Table S1. *Reasons for the 33 failures of LifeSoaks to predict the binding site bottleneck radii in the dataset of true positive soaking examples.*

PDB-ID	Ligand Identifier	Fail Reason
1ckj	WO4_A_400	unknown valence state
1ckj	WO4_A_401	unknown valence state
1ckj	WO4_B_400	unknown valence state
1ckj	WO4_B_401	unknown valence state
1ckj	WO4_B_402	unknown valence state
1fdi	NO2_A_804	covalent bond to protein residue
1o01	CRD_C_4513	covalent bond to protein residue
1o01	CRD_F_4516	covalent bond to protein residue
1o01	CRD_G_4517	covalent bond to protein residue
1zqe	CR_A_340	could not be initialized
1zqe	CR_A_341	could not be initialized
2rbk	VN4_A_601	unknown valence state
3bca	IOD_A_520	covalent bond to protein residue
3bca	IOD_A_528	covalent bond to protein residue
3bca	IOD_A_537	covalent bond to protein residue
3oib	IOD_B_512	covalent bond to protein residue
4akm	IR3_A_1378	could not be initialized
4akm	IR3_A_1380	could not be initialized
4akm	IR3_B_1379	could not be initialized
4akm	IR3_B_1381	could not be initialized
4f3a	IR3_A_601	could not be initialized
4hgo	VN4_A_201	unknown valence state
4hgo	VN4_B_201	unknown valence state
4hgo	VN4_C_201	unknown valence state
4hgo	VN4_D_202	unknown valence state
4hgp	VN4_A_202	unknown valence state
4lsh	BR_B_404	covalent bond to protein residue
4lsi	BR_A_410	covalent bond to protein residue
4pwc	BR_A_626	covalent bond to protein residue
4r15	CR_A_101	could not be initialized
4r15	CR_A_102	could not be initialized
4r15	CR_B_101	could not be initialized
5n5q	FFE_A_202	unknown valence state

S6 Reasons for false negative predictions

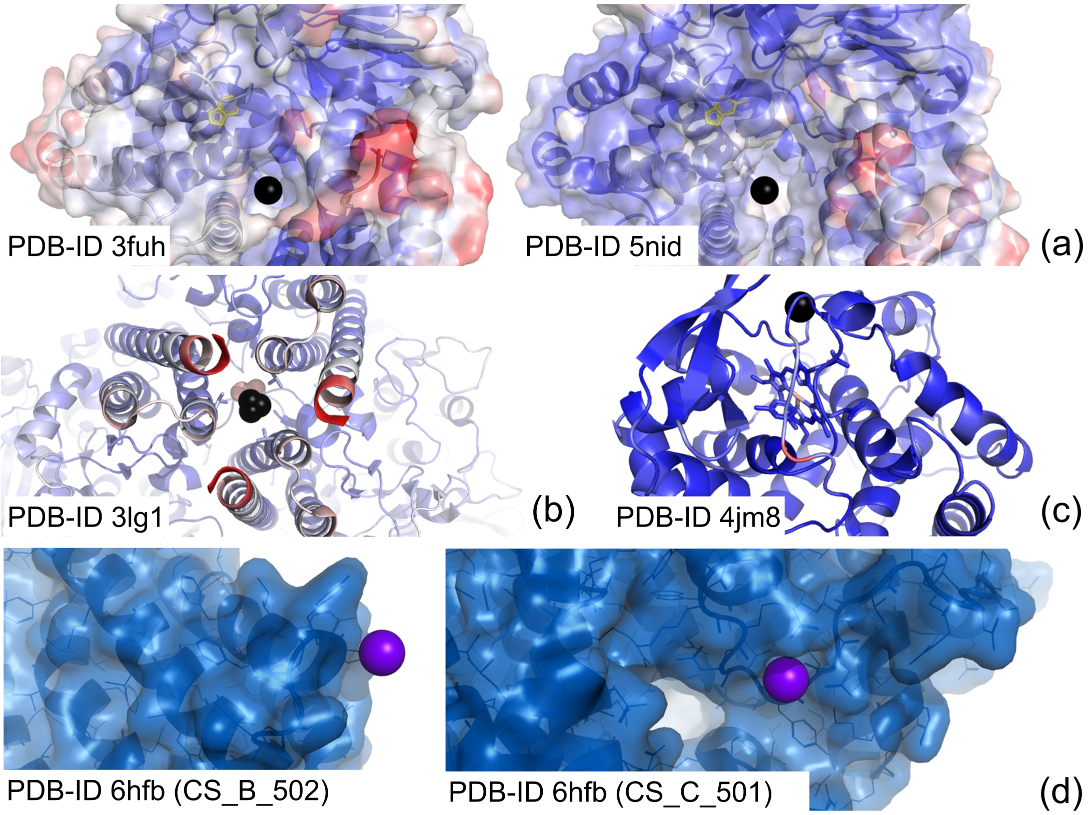

Fig. S4. Showcases providing explanations for false negative predictions with *LifeSoaks*. (a) Domain flexibility of leukotriene A4 hydrolase might explain the inaccessibility of the binding site as analyzed for the soaked structure. In the protein structure with the PDB-ID 5nid, the binding site is predicted as accessible. (b) The structure of cytochrome C nitrite reductase (PDB-ID 3lg1) is characterized by flexible helix termini in the proximity of the bottleneck to the binding site of the sulfite ion (SO_3^{2-}, PDB three-letter code SO3 in chain A with residue ID 537). This protein conformation might open and close upon ligand binding. (c) Structures with low electron density support for binding site-enclosing regions might lead to false negative results, as shown for cytochrome C peroxidase (PDB-ID 4jm8). (d) Binding site bottleneck radii for highly solvent-exposed binding sites cannot be correctly predicted and are out of the method's scope (as shown in the example with the PDB-ID 6hfb).

S7 1D, 2D, and 3D bottleneck radii predicted by *LifeSoaks* and *MAP CHANNELS*

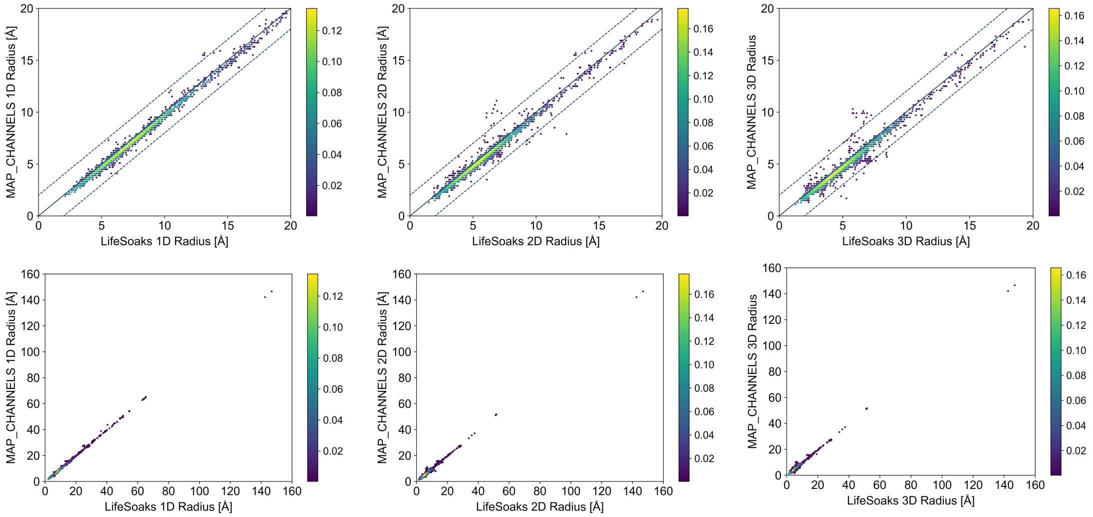

Fig. S5. 1D, 2D, and 3D bottleneck radii for the dataset of true positive soaking examples as calculated by *LifeSoaks* and *MAP_CHANNELS*. The top figures show scatter plots with of crystal structures with bottleneck radii up to 20 Å while the bottom figures show the distributions of all bottleneck radii in the dataset. The points are colored according to the kernel-density estimate using Gaussian kernels visualizing highly occupied regions in the dataset.
